# Sugary drink warnings: A meta-analysis of experimental studies

**DOI:** 10.1371/journal.pmed.1003120

**Published:** 2020-05-20

**Authors:** Anna H. Grummon, Marissa G. Hall

**Affiliations:** 1 Harvard Center for Population and Development Studies, Harvard T.H. Chan School of Public Health, Cambridge, Massachusetts, United States of America; 2 Department of Population Medicine, Harvard Medical School and Harvard Pilgrim Health Care Institute, Boston, Massachusetts, United States of America; 3 Department of Health Behavior, University of North Carolina Gillings School of Global Public Health, Chapel Hill, North Carolina, United States of America; 4 Lineberger Comprehensive Cancer Center, University of North Carolina at Chapel Hill, Chapel Hill, North Carolina, United States of America; 5 Center for Health Promotion and Disease Prevention, University of North Carolina at Chapel Hill, Chapel Hill, North Carolina, United States of America; University of Cambridge, UNITED KINGDOM

## Abstract

**Background:**

Policymakers worldwide are considering requiring warnings for sugary drinks. A growing number of experimental studies have examined sugary drink warnings’ impacts, but no research to our knowledge has synthesized this literature. To inform ongoing policy debates, this study aimed to identify the effects of sugary drink warnings compared with control conditions.

**Methods and findings:**

We systematically searched 7 databases on June 21, 2019, and October 25, 2019. We also searched reference lists of relevant articles. Two investigators independently screened titles, abstracts, and full texts to identify peer-reviewed articles that used an experimental protocol to examine the effects of sugary drink warnings compared to a control condition. Two investigators independently extracted study characteristics and effect sizes from all relevant full-text articles. We meta-analyzed any outcome assessed in at least 2 studies, combining effect sizes using random effects meta-analytic procedures. Twenty-three experiments with data on 16,241 individuals (mean proportion female, 58%) were included in the meta-analysis. Most studies took place in Latin America (35%) or the US or Canada (46%); 32% included children. Relative to control conditions, sugary drink warnings caused stronger negative emotional reactions (*d* = 0.69; 95% CI: 0.25, 1.13; *p* = 0.002) and elicited more thinking about the health effects of sugary drinks (*d* = 0.65; 95% CI: 0.29, 1.01; *p <* 0.001). Sugary drink warnings also led to lower healthfulness perceptions (*d* = −0.22; 95% CI: −0.27, −0.17; *p <* 0.001) and stronger disease likelihood perceptions (*d* = 0.15; 95% CI: 0.06, 0.24; *p* = 0.001). Moreover, sugary drink warnings reduced both hypothetical (*d* = −0.32; 95% CI: −0.44, −0.21; *p <* 0.001) and actual consumption and purchasing behavior (*d* = −0.17; 95% CI: −0.30, −0.04; *p* = 0.012). Statistically significant effects were not observed for perceptions of added sugar or positive sugary drink attitudes (*p*’s > 0.10). Moderation analyses revealed that health warnings (e.g., “Beverages with added sugar contribute to obesity”) led to greater reductions in hypothetical sugary drink purchases than did nutrient warnings (e.g., “High in sugar”; *d* = −0.35 versus −0.18; *Q*_b_ = 4.04; *p* = 0.04). Limitations of this study include that we did not review grey literature and that we were unable to conduct moderation analyses for several prespecified moderators due to an insufficient number of studies.

**Conclusions:**

This international body of experimental literature supports sugary drink warnings as a population-level strategy for changing behavior, as well as emotions, perceptions, and intentions.

**Protocol Registry:**

PROSPERO ID 146405.

## Abstract 2

Anna Grummon and colleagues reveal the potential of warnings labels on sugary drinks to deter consumption.

## Author summary

**Why was this study done?:**

- Policies requiring warnings for sugary drinks could discourage sugary drink consumption and reduce diet-related disease.
- A growing number of experimental studies have examined consumers’ responses to sugary drink warnings, but no research to our knowledge has synthesized these studies, making it difficult for policymakers to anticipate the impacts of sugary drink warning policies.

**What did the researchers do and find?:**

- Using a comprehensive search strategy, we identified and meta-analyzed 23 experiments, representing more 16,000 individuals, that assessed the impact of sugary drink warnings versus control conditions.
- We found that sugary drink warnings reduced both self-reported and objectively measured purchases of sugary drinks.
- Warnings also led to beneficial changes in mechanisms underlying longer-term behavior change, including causing stronger emotional responses, increasing perceptions that sugary drinks contribute to disease, and reducing intentions to purchase or consume sugary drinks.

**What do these findings mean?:**

- Requiring warnings for sugary drinks is a promising policy strategy for informing consumers and reducing consumption of sugary drinks.
- Future research will clarify optimal warning design, the trajectory of warnings’ effects over time, and warnings’ impacts on key population subgroups.

## Introduction

Consumption of sugary drinks (also known as sugar-sweetened beverages) remains a major public health problem globally [1–3]. Among unhealthy products, sugary drinks are often singled out as particularly problematic because they are high in sugar and calories but low in nutrients [4], and because sugary drink consumption is associated with increased risk of many of the most pressing public health problems, including weight gain, obesity, dental caries, type 2 diabetes, and heart disease [5–9]. While individually delivered nutrition education interventions such as workshops, text messages, and meetings with dieticians can yield small reductions in sugary drink intake among those they reach [10], experts agree that population-level strategies are urgently needed to achieve meaningful, population-wide reductions in sugary drink consumption [3,11,12]. Compared to interventions delivered to individuals, population-wide policies have high reach, rely less on individual motivation to participate, and are often more cost-effective [13]. One promising policy for reducing sugary drink consumption is requiring warnings, for example on packaging or at the point of sale.

**Fig 1:**
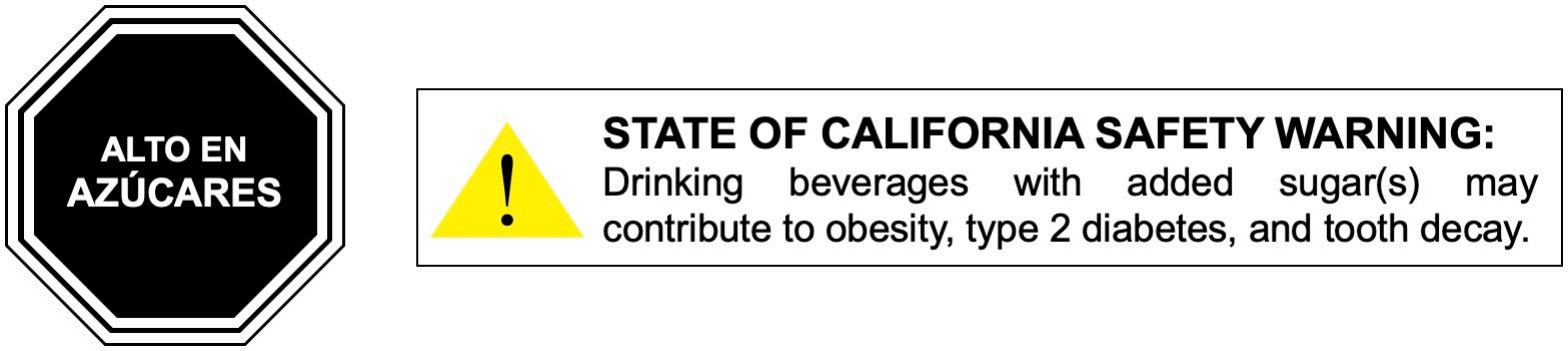
Sugary drink nutrient warning and health warning. The warning on the left is a nutrient warning based on warnings implemented in Chile in 2016; the text translates to “HIGH IN SUGARS.” The warning on the right is a health warning based on the warning proposed in California in 2019.

Food and beverage warning policies are increasingly popular globally. Two types of warnings have been proposed: nutrient warnings (messages that alert consumers that a food or beverage has a high amount of a harmful nutrient) and health warnings (messages that describe health harms of a particular product) (Fig 1). In 2016, Chile became the first country to require nutrient warnings to appear on sugary drinks and other energy-dense, nonessential foods that exceed recommended levels of added sugar, saturated fat, sodium, or calories [14]. Similar nutrient warning policies have been passed or implemented in Peru, Uruguay, Mexico, and Israel, and are under consideration in Brazil, Canada, and South Africa [15]. In the US, lawmakers in 5 states [16–20] have proposed laws requiring health warnings on sugary drink packaging, on vending machines, and at the point of sale of unsealed drinks, and one municipality (San Francisco) has passed an ordinance requiring health warnings on sugary drink advertisements (although the ordinance has not yet gone into effect due to industry litigation) [21].

Research on the impacts of sugary drink warnings is urgently needed to guide policymakers considering implementing new sugary drink warning policies or improving existing regulations. As sugary drink warnings have gained traction among policymakers, a growing literature has begun to examine warnings’ impacts. Experimental studies are one useful method for studying warnings’ impacts because experiments provide strong evidence on the causal effect of sugary drink warnings on consumer behavior as well as key predictors of long-term behavior change. To date, no research to our knowledge has synthesized the experimental literature on sugary drink warnings. Thus, to inform active policy debates and future research on sugary drink warnings, the objective of this study was to meta-analyze randomized experiments examining the impacts of sugary drink warnings. Our primary research question was, across the body of experimental studies, what are the effects of sugary drink warnings compared with control conditions?

## Methods

Prior to data extraction, we pre-registered our search strategy, inclusion and exclusion criteria, and analysis plans on PROSPERO (ID 146405; see also S1 Text). We made 1 modification to the pre-registered analytic plan: While we planned to meta-analyze any outcome assessed in at least 2 studies, we did not meta-analyze perceptions of calorie content in sugary drinks (assessed in 2 studies [22,23]) given that we included calorie content labels as a relevant comparator condition. This meta-analysis used de-identified secondary data only and was exempt from human subjects review.

### Search strategy

We adhered to the guidelines of the Preferred Reporting Items for Systematic Reviews and Meta-Analyses (PRISMA) Statement [24] (S1 Table). To identify relevant studies, we implemented a comprehensive search strategy developed in collaboration with an academic research librarian. The search strategy comprised 2 steps. First, we searched 7 databases (PubMed, Scopus, Cochrane Central Register of Controlled Trials, Embase, CINAHL, PsycINFO, and Communication & Mass Media Complete) using the following terms and their cognates and synonyms: beverage AND (warning OR label OR message OR claim) (S2 Table contains details on search terms and results). We implemented an initial database search on June 21, 2019, and updated the search on October 25, 2019. The search included articles published at any time and in any language. Second, we examined the reference sections of the final set of articles included in the review.

### Inclusion and exclusion criteria

We included articles published in any year that used an experimental protocol to test sugary drink warnings compared to control conditions (Table 1). Experimental designs could use between-person manipulations (participants randomized to different conditions) or within-person manipulations (participants exposed to multiple conditions in a random order).

**Table 1:** Inclusion and exclusion criteria.

Category	Included	Excluded
Population	Individuals of any age	Studies not done in humans (e.g., animal studies)
Intervention	Any warning, with or without icons or pictures, intended to be displayed (i.e., designed or possible to be displayed) on the front of package of a sugary drink container, on a menu, or at the point of sale of sugary drinks, including (1) health warnings (messages that make a direct statement about health harms of consuming a nutrient or product); (2) nutrient warnings (messages that alert consumers that a product has a high amount of a harmful nutrient [i.e., sugar, salt, fat, saturated fat, trans fat, or calories] using words such as “high in” or “excess”); or (3) health and nutrient warnings (messages that include both health and nutrient warnings)	Labels that are not health or nutrient warnings, including (1) traffic light labels, Health Star Rating, or Facts Up Front labels; (2) calorie, nutrient, or “energy content” labels that display numerical content of a nutrient (e.g., 150 calories) without the words signaling excessive content; (3) labels with symbols only and no text; (4) health or nutrient claims meant to encourage consumption of the product (e.g., “high fiber”); (5) labels or warnings shown on advertisements; or (6) other communication interventions (e.g., text messages, public service announcements) or multicomponent interventions that do not separately report results of warnings’ impact
Comparators	(1) No-label control condition; (2) neutral images (e.g., barcode label); (3) neutral text statements (e.g., messages about littering); or (4) calorie labels or nutrient content labels including Facts Up Front labels and Guideline Daily Amount labels as long as they are not color-coded with a traffic light schema and do not signal high levels of unhealthy nutrients using phrases such “high in”	(1) Traffic light labels; (2) Health Star Rating; (3) health or nutrient content claims meant to encourage consumption of the product (e.g., “high fiber”); (4) nutrient content labels that include photographs or icons displaying nutrient content; or (5) no relevant control group (studies in which warnings are compared to one another)
Product	Sugary drinks, including sodas, sports drinks, fruit drinks, sweetened teas, sweetened coffees, flavored milks, and juice	Alcoholic beverages, foods, cigarettes, or other tobacco products
Outcomes	Any measured outcome, including purchase behaviors, intentions, healthfulness perceptions, product attitudes, and warning reactions; effect sizes were calculated for any outcome assessed in 2 or more studies	NA
Timing	Studies published anytime	NA
Setting	Any country	NA
Study design	Studies that are (1) true experiments (random assignment to conditions or random ordering of within-person conditions, including Latin square randomization) and (2) peer-reviewed original articles	Studies that are (1) not true experiments (e.g., quasi-experiments, natural experiments, or observational studies); (2) exact duplicate publications or studies publishing on exact duplicate data; (3) review articles or meta-analyses; (4) not peer-reviewed (dissertations, reports); (5) clinical case reports; (6) policy briefs or position statements; or (7) posters or conference abstracts
Language	Studies published in any language, as long as the full text can be translated into English	Studies published in languages other than English if the full text is not available to be translated into English

NA, not applicable.

To be included, studies had to report data on both a relevant warning condition and a relevant control condition. Relevant warnings were any sugary drink warning, with warnings defined as messages that made a direct statement about the product’s health effects (e.g., “contributes to obesity”), alerted consumers that a product contains an excessive amount of an unhealthy nutrient (e.g., a stop sign logo with the statement “high in sugar”), or both. To be eligible interventions, warnings had to be intended to be displayed on products’ front of package, on a menu, or at the point of sale. We excluded studies of warnings intended for advertisements because the impact of warnings on ads could be distinct from the impact in other settings due to the overwhelming presence of marketing elements on ads. Additionally, most sugary drink warning policies that have been proposed or implemented have required product and/or point-of-sale warnings [14,16–20]. Eligible warnings could be text only or could include a picture or icon.

Relevant control conditions included no-label conditions, neutral images or messages (e.g., a barcode label), and calorie or nutrient content labels without interpretive information (e.g., “240 calories per bottle”). We selected calorie and nutrient content labels as relevant comparators given that many products already display this information on the front of package. We did not consider traffic light labels, health or nutrient content claims meant to encourage consumption of a product (e.g., “high in fiber”), or Health Star Rating labels to be relevant comparators because these labels are designed to convey qualitative information about risk or healthfulness. S3 Table provides detailed definitions and examples of relevant interventions and comparators.

Studies could assess any outcome. We excluded studies if they were not original research (e.g., review articles), if they were not peer-reviewed, or if they were exact duplicate publications or used exact duplicate data.

### Article selection

Two investigators independently screened titles using Zotero (Corporation for Digital Scholarship, Vienna, Virginia, US); any title retained by at least 1 investigator was further screened. Next, 2 investigators independently screened abstracts and full-text articles using the online software Covidence (Veritas Health Innovation, Melbourne, Victoria, Australia). Discrepancies in abstract and full-text screening were resolved by a third investigator.

### Article coding

#### Coding study characteristics

Two investigators independently extracted study characteristics including publication year, country where data collection took place, sample characteristics (e.g., mean age), type of randomization (between-person versus within-person), number of warning exposure sessions, exposure setting for warnings/products (e.g., computer survey), exposure medium for warnings (e.g., front of package), characteristics of each warning condition (e.g., warning topic: health versus nutrient versus combined), and characteristics of each control condition (e.g., type of control). Discrepancies in study characteristic extraction were resolved by a third independent reviewer.

#### Coding dependent variables

Based on an initial review of relevant studies, we developed a list of more than 50 dependent variables measured in the studies. We then grouped these dependent variables into theory- or policy-relevant constructs. Table 2 describes the constructs assessed in at least 2 studies. We grouped these constructs into 6 categories, guided by the Message Impact Framework used in a previous meta-analysis of tobacco warnings [25]. The first group, titled “behavior,” included real-stakes (i.e., non-hypothetical) behavioral endpoints such as sugary drink purchases in a shopping task. Second, “attention and noticing” included participants’ noticing of or attention to warnings. Third, “warning reactions” assessed participants’ emotional and cognitive responses to warnings (e.g., extent to which warnings elicit fear) [25]. Fourth, “attitudes and beliefs” included participants’ attitudes toward and beliefs about sugary drinks and sugary drink consumption (e.g., perceptions that sugary drinks are healthy). Fifth, “intentions and hypothetical choices” examined self-reported hypothetical purchases, self-reported likelihood of buying or consuming sugary drinks, and self-reported selection of beverage coupons. Finally, we also assessed “policy support,” which examined participants’ support of policies requiring sugary drink warnings. We did not differentiate between objectively measured versus self-reported outcomes for any constructs other than behavior because none of the meta-analyzed studies objectively measured non-behavioral outcomes.

**Table 2:** Outcomes assessed in meta-analysis of experimental studies of sugary drink warnings.

Construct	Definition	Example item	Example(s) of authors’ terminology
**Behavior**			
Sugary drink purchase behavior (primary outcome)	Participants’ selection or purchase of sugary drinks in non-hypothetical shopping or choice scenarios	NA (objectively measured)	Sugar-sweetened beverage purchases, percent purchased sugary drink
Calories purchased from beverages	Total calories participants purchased from beverages in non-hypothetical shopping or choice scenarios	NA (objectively measured)	Calories purchased
Grams of sugar purchased from beverages	Total sugar participants purchased from beverages in non-hypothetical shopping or choice scenarios	NA (objectively measured)	Free sugar purchased
**Attention and noticing**			
Noticed nutrition or trial label	Whether participants report noticing nutrition or trial label(s)	“In all of the previous purchasing tasks, did you notice any nutrition labels or symbols on the front of the food and beverage packages?” [26]	Noticing of FOP label, noticed trial label
**Warning reactions**			
Negative emotional reactions	Negative emotional responses to warnings such as worry, fear, or disgust	“How worried does this image make you feel?” [27]	Negative emotions, negative mood, negative emotional arousal
Thinking about the health effects of sugary drinks	Extent to which participants report thinking about the health effects of sugary drinks	“How much did the labels make you think about the health problems caused by drinking beverages with added sugar?” [28]	Health consideration, cognitive elaboration, thinking about harms
**Attitudes and beliefs about sugary drinks**			
Healthfulness perceptions	Perception that sugary drinks are (or consuming sugary drinks is) healthy	“How healthy do you think this product is?” [23]	Perceived healthfulness ratings, product healthfulness
Positive outcome expectancies	Beliefs that consuming sugary drinks will result in positive outcomes	“Drinking this product often would make you feel energized.” [22]	Focus, energized
Positive product attitudes	Positive evaluation of sugary drinks	“Say how unappealing or appealing you think each beverage is.” [28]	Product attractiveness, product appeal, coolness, deliciousness
Perceived disease likelihood	Beliefs that consuming sugary drinks is likely to lead to disease or health-related harms	“Drinking this product often would increase your risk of diabetes.” [22]	Risk perceptions, sugar-sweetened beverage disease risk, perceived health risks
Perceptions of amount of added sugar	Perceptions of the amount of added sugar in sugary drinks	“How much added sugar do you think is in this 20-ounce bottle?” [23]	Added sugar
**Policy support**			
Policy support	Extent to which participants would support policies requiring sugary drink warnings	“Do you support putting this label on sugar-sweetened beverages?” [29]	Consumer support, acceptability
**Intentions and hypothetical choices**			
Hypothetical purchases of sugary drinks	Participants’ selection or purchase of sugary drinks in hypothetical shopping or choice scenarios	NA (amount or selection in choice or shopping task)	Vending machine choice, selection of sugar-sweetened beverage in choice scenario
Purchase or consumption intentions	Likelihood of purchasing or consuming sugary drinks	“How likely are you to drink this product in the next 4 weeks?” [22]	Purchase likelihood
Hypothetical coupon selection—sugary drinks	Participants’ uptake of coupons for sugary drinks in hypothetical shopping or choice scenarios	“Indicate all beverages you would buy for your child for which you would like to receive a coupon.” [23]	Number of sugar-sweetened beverage coupons
Hypothetical coupon selection—non-sugary drinks	Participants’ uptake of coupons for non-sugary drinks in hypothetical shopping or choice scenarios	“Indicate all beverages you would buy for your child for which you would like to receive a coupon.” [23]	Number of non-sugar-sweetened beverage coupons
Hypothetical total expenditure on beverages	Participants’ total expenditures on beverages in hypothetical shopping or choice scenarios	NA (amount or selection in choice or shopping task)	Total expenditures on beverages

FOP, front-of-package; NA, not applicable.

**Fig 2:**
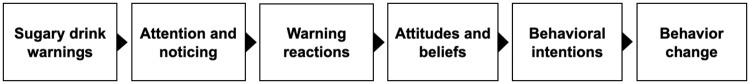
Conceptual model depicting sugary drink warnings’ impacts on behavior and psychological outcomes.

Fig 2 depicts our conceptual model of how these constructs relate to one another, developed using the Message Impact Framework [25] and previous studies of how warnings change behavior [29–33]. Briefly, our adapted Message Impact Framework suggests that sugary drink warnings will garner attention and noticing, which will elicit emotional and cognitive reactions to the warning messages. These reactions will then change attitudes and beliefs about sugary drinks, leading to increased intentions to reduce sugary drink purchases and consumption and, finally, to behavior change. Policy support, although of considerable interest to policymakers and advocates, is not included in the conceptual model because it is unlikely to be a driver of behavior change.

#### Effect size extraction and calculation

The 2 principal investigators independently extracted effect sizes, with discrepancies resolved by discussion. We characterized the effect size of the impact of sugary drink warnings compared to control conditions by using the standardized mean difference statistic *d*. We independently converted all effect size estimates into *d*’s using Stata’s effect size commands (when means and SDs were reported) or the online tool Practical Meta-Analysis Effect Size Calculator [34] (when other measures of effect were reported), resolving discrepancies by discussion. We contacted authors when articles did not report sufficient information to calculate standardized effect sizes; all authors replied and provided the requested data. (We did not meta-analyze Arrua and colleagues’ discrete choice experiment [35], as this design does not allow calculation of standardized effect sizes.) When studies reported multiple effect estimates for the same outcome (e.g., 2 measures of the effect of warnings on intentions), we combined effect sizes prior to analysis following methods described in Borenstein et al. [36] and assuming a correlation of 0.0 (primary analysis) or 0.5 (sensitivity analysis) among outcomes. The direction of effects and pattern of statistical significance were identical in these 2 analyses (see S4 Table), so we retained the analysis assuming a correlation of 0.0 as the primary analysis. When studies reported results for more than 1 relevant warning or control condition, we averaged effect sizes across relevant comparisons, adjusting for correlation among effect sizes using the formulae provided by Borenstein et al. [36]. Because *d* can be upwardly biased in studies with small samples [37], we applied Hedges’s correction for this bias to the extracted *d*’s, again using the formulae provided in Borenstein et al. [36].

### Quantitative synthesis and meta-analysis

We combined the *d*’s with Hedges’s corrections from individual studies using random effects meta-analysis, calculating between-study variance using the empirical Bayes/Paule–Mandel method [38–40]. Our prespecified primary outcome was real-stakes (i.e., non-hypothetical) purchases or selection of sugary drinks. We also meta-analyzed any outcome with usable effect sizes from 2 or more studies (see above for the 1 exception, perceptions of calorie content). For each meta-analyzed outcome, we report the mean weighted effect size as well as its 95% confidence interval (CI), interpreting CIs that did not overlap 0 as statistically significant effects. We assessed heterogeneity using the *I*^2^ and *Q* statistics.

We planned to conduct moderation analyses for 5 prespecified key outcomes with policy and public health relevance: real-stakes purchases of sugary drinks, purchase or consumption intentions, hypothetical purchases, perceived disease likelihood, and healthfulness perceptions. For these outcomes, we conducted moderation analyses when significant heterogeneity existed among effect sizes and effect sizes were available from at least 2 studies per level of the moderator. We examined whether effect sizes differed by the following prespecified categorical moderators: warning topic (health versus nutrient), sugary drink consumer status of sample (all sugary drink consumers versus not all sugary drink consumers, including studies that did not report consumption status), and ages included in sample (includes children aged <18 years versus does not, including studies that did not report whether children were included). For the moderation analyses examining hypothetical purchases by warning topic, we used 2 effect sizes from Ang et al. [41], 1 for health warning versus control and 1 for nutrient warning versus control. For these 2 effect sizes, we partitioned the control group sample size equally across the 2 treatment arms, following others [42,43]. This approach partially (but not completely) corrects for correlation among effect sizes, so this moderation analysis should be interpreted with caution [43]. For all moderation analyses, we calculated effect sizes and 95% confidence intervals for each level of the moderating variable and assessed heterogeneity in those effect sizes using the *Q*_b_ statistic. All analyses used Stata version 16 (StataCorp, College Station, Texas, US).

## Results

### Article selection

**Fig 3:**
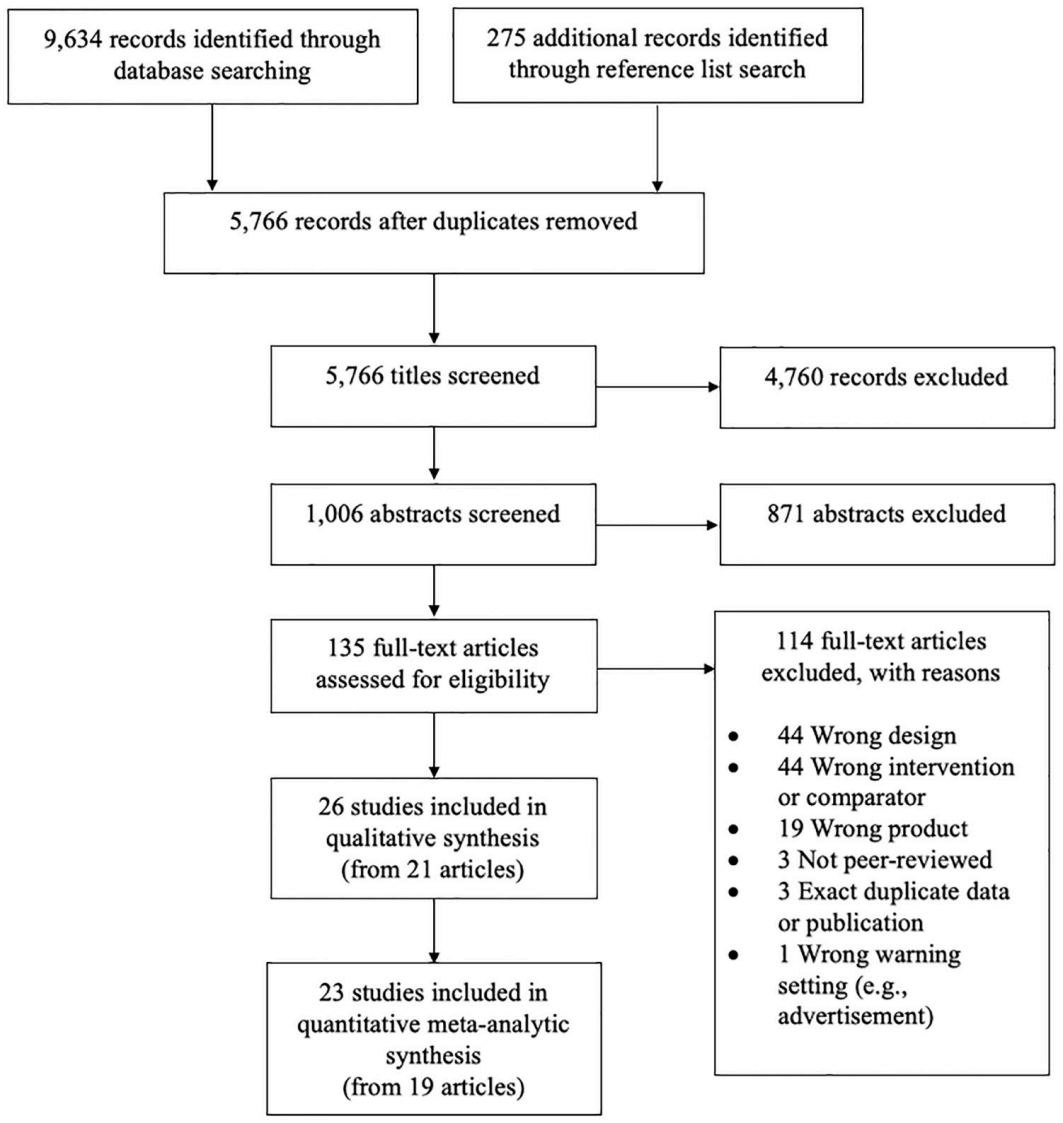
PRISMA flow diagram.

As detailed in Fig 3, the database searches yielded 9,634 records before excluding duplicates. Reference list searches of included articles yielded an additional 275 records, for a total of 5,766 records after excluding duplicates. During title screening, 4,760 of these records were excluded. Of the remaining 1,006 records, 871 were excluded based on abstract screening, leaving 135 full-text articles assessed for eligibility. Twenty-one of these full-text articles (reporting on 26 individual experimental studies) were eligible for inclusion in the qualitative synthesis [22,23,26–29,35,41,44–56]. Of these, 19 articles (reporting on 23 individual experiments and representing 16,241 individuals) were included in the quantitative meta-analysis. S5 Table provides details on relevant studies.

### Study characteristics

All 26 studies were published in 2016 or later, with 81% published in 2018 or later (Table 3). Most studies were conducted in the United States or Canada (46%) or Latin American (35%). Most studies (69%) displayed warnings via a computer survey. Three studies (12%) displayed warnings in a naturalistic laboratory store or pantry, and no studies displayed warnings in an actual store or restaurant environment. About two-thirds (62%) of studies examined health warnings, 50% examined nutrient warnings, and 8% examined combined health and nutrient warnings. The majority (77%) of studies examined text-only warnings, 31% examined warnings with pictures, and 1 study examined warnings with icons. The majority (69%) of studies displayed warnings on the front of package of a sugary drink container. All studies used a single exposure session. The most common type of comparator used was a no-label control condition (77% of studies), followed by calorie or nutrient content labels (31%). Most studies (73%) used between-person randomization. Most studies (92%) included adults; 7 studies included children, comprising 32% of the 22 studies reporting the age range included in the sample.

**Table 3:** Characteristics of studies and study samples (*k* = 26).

Characteristic	Percent or mean	*k* or SD
**Characteristics of studies**		
Year study published, percent of studies		
2016–2017	19%	5
2018–2019	81%	21
Region, percent of studies		
US or Canada	46%	12
Latin America	35%	9
Europe or Oceania	15%	4
Asia	4%	1
Setting in which warning or product displayed, percent of studies		
Computer survey	69%	18
Paper survey	8%	2
Naturalistic online store	8%	2
Naturalistic laboratory store or pantry	12%	3
Projected on a screen	4%	1
Actual store	0%	0
Warning topic(s) studied^a^, percent of studies		
Health warning	62%	16
Nutrient warning	50%	13
Health and nutrient warning	8%	2
Warning type(s) studied^a^, percent of studies		
Text only	77%	20
Graphic	31%	8
Icon	4%	1
Exposure medium, percent of studies		
Warning on front of package	69%	18
Warning above/below/next to image of product	19%	5
Warning by itself	8%	2
Warning shown before image of product	4%	1
Number of exposure sessions, percent of studies		
1 session	100%	26
2 or more sessions	0%	0
Comparator(s) used^a^, percent of studies		
No-label control	77%	20
Calorie or nutrient content label	31%	8
Neutral message or image	12%	3
Type of randomization used for primary comparison, percent of studies		
Between-person	73%	19
Within-person	27%	7
**Characteristics of study samples**		
Mean age, years^b^	31.5	9.8
Mean age not reported, percent of studies	46%	12
Age ranges included, percent of studies		
Children (0–17 years) included^c^	32%	7
Adults (18+ years) included	92%	24
Sample’s sugary drink consumption status, percent of studies		
Sugary drink consumers only	15%	4
Mix of sugary drink consumers and non-consumers	35%	9
Did not report sugary drink consumption in sample	50%	13
Gender of sample, mean proportion in each category		
Women	0.58	0.12
Men	0.42	0.12

^a^Categories sum to >100% because studies could examine more than 1 category.

^b^Among studies reporting mean age (*k* = 14).

^c^Among studies that reported whether or not sample included children (*k* = 22).

*k*, number of studies.

### Effects of sugary drink warnings

#### Behavior

**Fig 4:**
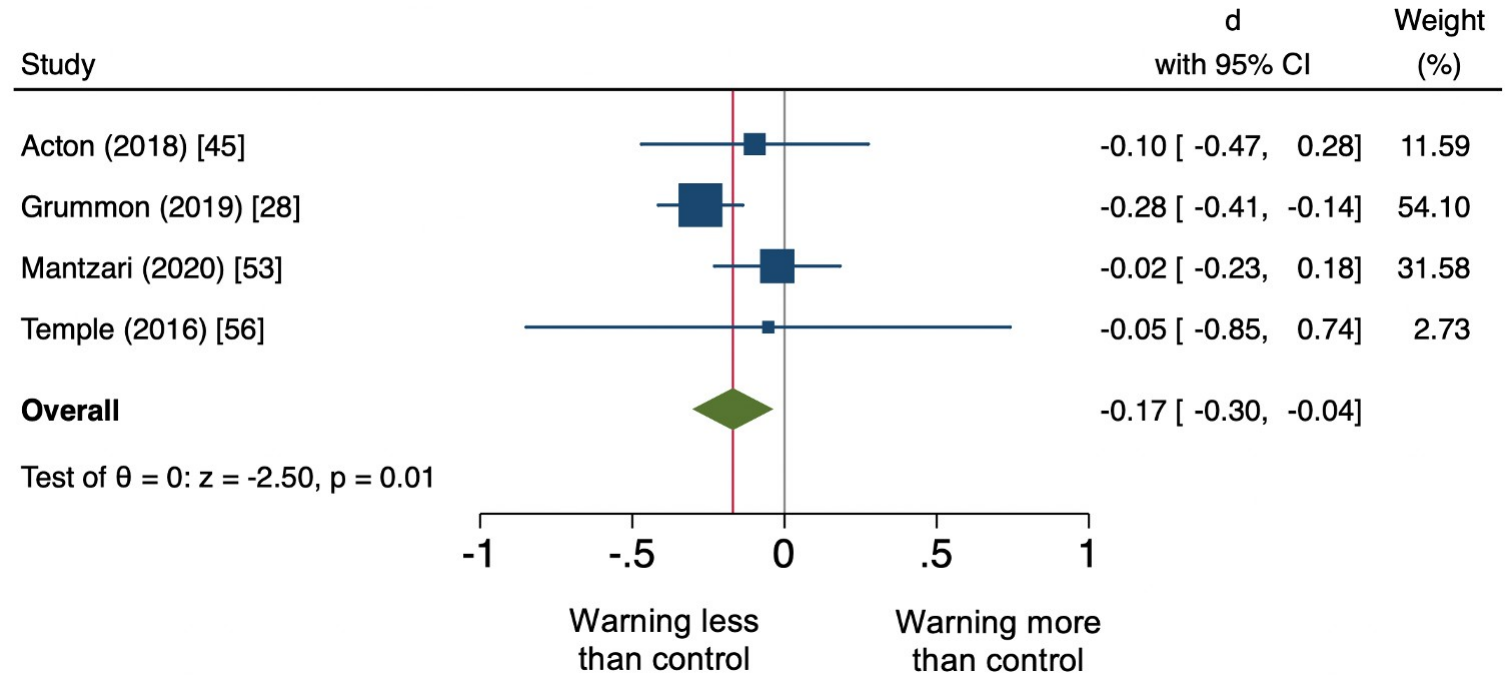
Forest plot displaying effect sizes and 95% CIs for real-stakes selection or purchases of sugary drinks (primary outcome). The plot shows effect sizes (Cohen’s *d* with Hedges’s correction; represented by the midpoint of each box) and 95% confidence intervals (represented by the width of each box) for each meta-analyzed study. The weight given to each study in the meta-analysis is listed in the final column and is represented by the area of the box. The overall (meta-analytic) effect size and its confidence interval are represented by the midpoint and width of the diamond, respectively.

Meta-analysis revealed that sugary drink warnings led to beneficial effects for all 3 real-stakes behavioral endpoints (Table 4). Fig 4 shows the forest plot of the 4 experimental studies examining sugary drink purchase or selection behavior [28,45,53,56], the primary outcome. In meta-analysis of these studies, warnings led to lower purchases or selection of sugary drinks compared to control conditions (*d* with Hedges’s correction = −0.17; 95% CI: −0.30, −0.04). Sugary drink warnings also led to fewer calories purchased from beverages (*d* = −0.16; 95% CI: −0.24, −0.07; S1 Fig) and fewer grams of sugar purchased from beverages (*d* = −0.11; 95% CI: −0.21, −0.01; S2 Fig).

**Table 4:** Effects of sugary drink warnings versus control: Mean weighted effect sizes (*d* with Hedges’s correction) and heterogeneity statistics (*k* = 23 studies), primary analyses.

Outcome	*N*	*k*	*d* (95% CI)	*Q*	*p*	*I*^2^
**Behavior**						
Purchases of sugary drinks (primary outcome)	1,407	4	**−0.17 (−0.30, −0.04)**	4.30	0.231	16.61
Calories purchased from beverages	2,338	3	**−0.16 (−0.24, −0.07)**	1.23	0.540	0.01
Grams of sugar purchased from beverages	1,938	2	**−0.11 (−0.21, −0.01)**	0.00	0.949	0.00
**Attention and noticing**						
Noticed nutrition or trial labels	1,840	2	**0.83 (0.54, 1.12)**	6.36	0.012	84.29
**Warning reactions**						
Negative emotional reactions	3,594	4	**0.69 (0.25, 1.13)**	137.52	<0.001	97.39
Thinking about the health effects of sugary drinks	2,543	4	**0.65 (0.29, 1.01)**	41.54	<0.001	93.69
**Attitudes and beliefs about sugary drinks**						
Healthfulness perceptions	6,947	9	**−0.22 (−0.27, −0.17)**	8.15	0.419	1.68
Positive outcome expectancies	4,583	2	**−0.26 (−0.34, −0.17)**	3.93	0.047	74.55
Positive product attitudes	5,969	6	−0.54 (−1.43, 0.35)	223.34	<0.001	99.74
Perceived disease likelihood	7,072	6	**0.15 (0.06, 0.24)**	39.46	<0.001	83.02
Perceptions of amount of added sugar	4,983	3	0.25 (−0.05, 0.55)	38.55	<0.001	95.37
**Policy support**	2,132	2	0.19 (−0.14, 0.51)	13.86	<0.001	92.79
**Intentions and hypothetical choices**						
Hypothetical purchases of sugary drinks	7,681	6	**−0.32 (−0.44, −0.21)**	14.70	0.012	78.69
Purchase or consumption intentions	7,118	8	**−0.30 (−0.44, −0.15)**	40.85	<0.001	89.36
Hypothetical coupon selection—sugary drinks	4,583	2	**−0.31 (−0.37, −0.25)**	0.00	0.972	0.04
Hypothetical coupon selection—non-sugary drinks	4,583	2	−0.02 (−0.21, 0.17)	9.57	0.002	89.55
Hypothetical total expenditure on beverages	1,189	2	−0.08 (−0.21, 0.06)	0.46	0.495	0.00

*N*, number of participants; *k*, number of studies/effect sizes; *d*, corrected standardized mean difference (pooled effect size). Effect sizes in bold are statistically significant at *p* < 0.05.

#### Attention and noticing

We were able to meta-analyze 1 attention and noticing outcome, noticing of nutrition or trial labels. Across the 2 studies reporting on noticing [26,28], sugary drink warnings were more likely to elicit noticing of nutrition/trial labels than control conditions (*d* = 0.83; 95% CI: 0.54, 1.12; S3 Fig).

#### Warning reactions

Sugary drink warnings showed advantages over control conditions in eliciting cognitive and emotional responses. Relative to control conditions, warnings elicited stronger negative emotional reactions (*d* = 0.69; 95% CI: 0.25, 1.13; S4 Fig) and more thinking about the health effects of sugary drinks (*d* = 0.65; 95% CI: 0.29, 1.01; S5 Fig).

#### Attitudes and beliefs about sugary drinks

Sugary drink warnings exhibited beneficial effects for 3 of the 5 attitudes and beliefs outcomes. Compared to control conditions, warnings led to lower perceptions of healthfulness of sugary drinks (*d* = −0.22; 95% CI: −0.27, −0.17; S6 Fig) and lower positive outcome expectancies (*d* = −0.26; 95% CI: −0.34, −0.17; S7 Fig). Sugary drink warnings also increased perceived disease likelihood (*d* = 0.15; 95% CI: 0.06, 0.24; S8 Fig). We did not observe statistically significant effects of sugary drink warnings on positive product attitudes (*d* = −0.54; 95% CI: −1.43, 0.35; S9 Fig) or perceptions of the amount of added sugar in sugary drinks (*d* = 0.25, −0.05, 0.55; S10 Fig), though these effect sizes were similar in magnitude to other outcomes.

#### Policy support

Two studies assessed warnings’ impacts on support for sugary drink policies. Meta-analysis of these studies did not find statistically significant effects of sugary drink warnings on policy support (*d* = 0.19; 95% CI: −0.14, 0.51; S11 Fig).

#### Intentions and hypothetical choices

Compared to control conditions, sugary drink warnings led to lower hypothetical purchases of sugary drinks (*d* = −0.32; 95% CI: −0.44, −0.21; S12 Fig), lower sugary drink purchase or consumption intentions (*d* = −0.30; 95% CI: −0.44, −0.15; S13 Fig), and lower hypothetical sugary drink coupon selection (*d* = −0.31; 95% CI: −0.37, −0.25; S14 Fig). Warnings did not exhibit statistically significant effects on hypothetical non-sugary drink coupon selection (*d* = −0.02; 95% CI: −0.21, 0.17; S15 Fig) and did not lead to statistically significant changes in hypothetical total expenditures on beverages (*d* = −0.08; 95% CI: −0.21, 0.06; S16 Fig).

### Moderation of sugary drink warnings’ impacts

Of the 5 prespecified outcomes we planned to examine in moderation analyses, 3 exhibited significant heterogeneity: purchase or consumption intentions (*Q* = 40.85; *p <* 0.001), hypothetical purchases of sugary drinks (*Q* = 14.70; *p* = 0.012), and perceived disease likelihood (*Q* = 39.46; *p <* 0.001) (Table 4). Moderation analyses of hypothetical purchases of sugary drinks found that the results of studies examining health warnings (*k* = 5) differed from those of studies examining nutrient warnings (*k* = 2) (S6 Table). While both warning topics led to lower hypothetical sugary drink purchases, health warnings had a larger impact (*d* for health warnings = −0.35; 95% CI: −0.47, −0.24; *d* for nutrient warnings = −0.18; 95% CI: −0.31, −0.05; *Q*_b_ = 4.04; *p* = 0.044). Moderation analyses found no differences by sugary drink consumer status of study samples in warnings’ effects on purchase or consumption intentions (*Q*_b_ = 0.54; *p* = 0.463) or perceived disease likelihood (*Q*_b_ = 0.10; *p* = 0.755). Likewise, warnings exerted similar effects on intentions among studies that did and did not include children (*Q*_b_ = 2.49; *p* = 0.115).

## Discussion

In this meta-analysis of experimental studies, sugary drink warnings exerted beneficial effects on real-stakes behavioral endpoints, including sugary drink purchases, calories purchased from beverages, and amount of sugar purchased from beverages. Sugary drink warnings also led to beneficial effects on noticing, emotions, thinking about health effects, several attitudes and beliefs, and behavioral intentions. The Message Impact Framework, along with health behavior and health communication theories, suggests that these changes in psychological outcomes are likely to promote longer-term behavior change, as depicted in Fig 2. Further, 2 recent randomized trials of tobacco [30,57] and sugary drink [31] warnings found that emotions, thinking about harms, and intentions were key mediators underlying warnings’ effects on behavior. Our results therefore suggest that implementing sugary drink warning policies could yield sustained changes in sugary drink consumption.

Warnings also influenced some attitudes and beliefs about sugary drinks, including reducing perceived healthfulness of sugary drinks and increasing perceptions that sugary drinks heighten disease likelihood. However, this study did not observe statistically significant effects of warnings on product attitudes or on perceptions of the added sugar content in sugary drinks, despite observing small to medium effect sizes for these outcomes. The lack of statistically significant impacts on these outcomes could reflect that studies often reported measures of attitudes about sugary drinks overall, rather than attitudes about specific types of beverages. A recent experimental study found that warnings’ impacts on beverage attitudes and perceptions varied across different types of sugary drinks (e.g., sodas versus sweetened teas) [58]; thus, some nuance may be lost in assessing warnings’ impacts on overall measures of attitudes and beliefs.

Questions remain about how to design sugary drink warnings to maximize their effectiveness. Latin American countries have opted for nutrient warnings while US jurisdictions have proposed health warnings. In our meta-analysis, both nutrient warnings and health warnings exerted beneficial effects. However, moderation analyses revealed that health warnings were more effective than nutrient warnings at lowering hypothetical purchases of sugary drinks. This finding is a preliminary indication that health warnings may be more impactful than nutrient warnings. However, we were unable to conduct similar moderation analyses by warning topic for real-stakes behavioral outcomes. Future research should continue to assess optimal warning design, particularly given that modest improvements in warning efficacy may yield large population-level health benefits [59,60].

Strengths of this study include that we implemented a comprehensive search across multiple databases coupled with reference list searches to identify articles not captured in database searches. Our search resulted in a large number of studies and a large overall sample size included in the analysis. We included only studies that used an experimental protocol to assess sugary drink warnings, yielding a sample of studies with high internal validity and at low risk of bias from confounding. Additionally, we examined studies of both health warnings and nutrient warnings, enabling us to assess the impacts of the 2 key types of warnings of interest to policymakers. We also meta-analyzed any outcome assessed in at least 2 studies, allowing us to provide a comprehensive analysis of sugary drink warnings’ impacts.

Limitations of this study include that we did not review grey literature, at the advice of an academic research librarian. While our decision to exclude grey literature ensures that our search can be repeated by other researchers and means that our review included only studies that had undergone peer review, we may have missed relevant non-peer-reviewed experimental studies of sugary drink warnings. We were also unable to conduct moderation analyses for several moderators due to an insufficient number of studies. We did not produce funnel plots to assess the potential for publication bias, given that these tests typically have low power [61] and that asymmetry may not reflect publication bias [62]. Finally, we did not conduct a formal risk of bias assessment; because this review focused on experimental studies, we excluded studies with high risk of bias to internal validity due to non-random assignment to study conditions.

Our review identified several key areas for future research. First, no experimental studies to our knowledge have examined sugary drink warnings over longer time periods. Warnings’ impacts on behavior might wane over time if consumers habituate to the messages [63]. Alternatively, sugary drink warnings’ impacts could be stable or even increase over time if warnings induce consumers to form new habits [64]. Longer-term studies are needed to clarify the trajectory of warnings’ impacts over time. Second, while several studies have used naturalistic laboratory settings, no experimental studies to our knowledge have been conducted in actual stores, cafeterias, or other real-world settings, limiting external validity. Future experimental studies should clarify consumers’ responses to sugary drink warnings in real-world settings. Such experimental studies would be an important complement to recent quasi-experimental research evaluating the real-world impact of Chile’s nutrient warnings on sugary drink purchases [65]. Relatedly, most outcomes included in our meta-analysis were assessed via self-report, and future studies should objectively measure other key mechanisms of behavior change [30,31], including attention (e.g., via eye tracking [55]) and emotional responses (e.g., via galvanic skin response or electromyography [66,67]). Third, a better understanding of how to design warnings is needed. While our meta-analysis suggests that health warnings may have advantages over nutrient warnings, more studies assessing both types of warnings’ impacts on behavior are needed, along with studies examining other warning characteristics (e.g., text versus pictorial warnings, inclusion of icons). Fourth, some potentially important outcomes were not assessed in sufficient studies to be meta-analyzed, including warning avoidance (which may promote warning efficacy [30,68]), stigma [50], social interactions [30,31,69,70], and purchases of foods and other types of beverages. Fifth, our meta-analysis focused on consumer responses to warnings, but warnings may also spark changes in industry behavior, such as product reformulation [71–73] or changes in advertising [74]. Natural and quasi-experimental evaluations of enacted warning policies are needed to assess industry responses to warnings. Finally, future studies should examine warnings’ effects on priority populations we were unable to study separately here. Our moderation analyses revealed that warnings’ effects on intentions did not vary based on the sugary drink consumer status of the sample or on inclusion of children in the sample, adding to a growing literature demonstrating that product warnings have similar effects across groups and therefore are unlikely to exacerbate disparities [22,23,28,57,75]. However, additional research is needed to clarify warnings’ effects on population groups most at risk for the health problems related to sugary drink consumption and to evaluate whether warnings could narrow underlying disparities in these outcomes.

Together, the findings in this meta-analysis support sugary drink warnings as a population-level strategy for reducing sugary drink purchases and eliciting psychological responses that underlie long-term behavior change. While warnings’ effects on behavioral outcomes were small in magnitude, simulation studies have found that reducing sugary drink intake by as little as 15–30 calories per day could reduce obesity prevalence by 1.5% to 7.8% and type 2 diabetes prevalence by up to 6.8% [59,76–80]. Our results suggest that policymakers should consider sugary drink warnings as a strategy for addressing overconsumption of sugary drinks and associated health harms.

## Supporting information

S1 Fig Forest plot displaying effect sizes and 95% CIs for calories purchased from beverages. (DOCX)

S2 Fig Forest plot displaying effect sizes and 95% CIs for grams of sugar purchased from beverages. (DOCX)

S3 Fig Forest plot displaying effect sizes and 95% CIs for noticing of nutrition or trial labels. (DOCX)

S4 Fig Forest plot displaying effect sizes and 95% CIs for negative emotional reactions. (DOCX)

S5 Fig Forest plot displaying effect sizes and 95% CIs for thinking about the health effects of sugary drinks. (DOCX)

S6 Fig Forest plot displaying effect sizes and 95% CIs for healthfulness perceptions. (DOCX)

S7 Fig Forest plot displaying effect sizes and 95% CIs for positive outcome expectancies. (DOCX)

S8 Fig Forest plot displaying effect sizes and 95% CIs for perceived disease likelihood. (DOCX)

S9 Fig Forest plot displaying effect sizes and 95% CIs for positive product attitudes. (DOCX)

S10 Fig Forest plot displaying effect sizes and 95% CIs for perceptions of amount of added sugar in sugary drinks. (DOCX)

S11 Fig Forest plot displaying effect sizes and 95% CIs for policy support. (DOCX)

S12 Fig Forest plot displaying effect sizes and 95% CIs for hypothetical purchases of sugary drinks. (DOCX)

S13 Fig Forest plot displaying effect sizes and 95% CIs for purchase or consumption intentions. (DOCX)

S14 Fig Forest plot displaying effect sizes and 95% CIs for hypothetical coupon selection—Sugary drinks. (DOCX)

S15 Fig Forest plot displaying effect sizes and 95% CIs for hypothetical coupon selection—Non-sugary drinks. (DOCX)

S16 Fig Forest plot displaying effect sizes and 95% CIs for hypothetical total expenditure on beverages. (DOCX)

S1 Table PRISMA checklist. (DOC)

S2 Table Search terms and results. (DOCX)

S3 Table Definitions of relevant interventions and comparators. (DOCX)

S4 Table Effects of sugary drink warnings versus control—Sensitivity analyses. (DOCX)

S5 Table Studies included in the review. (DOCX)

S6 Table Moderation of effects of sugary drink warnings versus control. (DOCX)

S1 Text PROSPERO registry record. (DOCX)
