# Supplementary material for: Sugary drink warnings: A meta-analysis of experimental studies
Source: PLoS Med. 2020 May 20;17(5):e1003120. doi: 10.1371/journal.pmed.1003120 (PMC7239392; doi:10.1371/journal.pmed.1003120)
Supplement: S1 Fig — (DOCX) [file pmed.1003120.s001.docx]

**S1 Fig.** Forest plot displaying effect sizes and 95% CIs for calories purchased from beverages.

Warning less than control

Warning more than control
