# Supplementary material for: Sugary drink warnings: A meta-analysis of experimental studies
Source: PLoS Med. 2020 May 20;17(5):e1003120. doi: 10.1371/journal.pmed.1003120 (PMC7239392; doi:10.1371/journal.pmed.1003120)
Supplement: S8 Fig — (DOCX) [file pmed.1003120.s008.docx]

**S8 Figure.** Forest plot displaying effect sizes and 95% CIs for perceived disease likelihood.

Warning more than control

Warning less than control
