# Supplementary material for: Sugary drink warnings: A meta-analysis of experimental studies
Source: PLoS Med. 2020 May 20;17(5):e1003120. doi: 10.1371/journal.pmed.1003120 (PMC7239392; doi:10.1371/journal.pmed.1003120)
Supplement: S2 Table — (DOCX) [file pmed.1003120.s018.docx]

**S2 Table.** Database searches and results.

| **Database** | **Search Strategy** | **Records returned** | |
| --- | --- | --- | --- |
|  |  | **6/21/2019** | **10/25/2019** |
| PubMed | (sugar sweetened beverage OR sugar sweetened beverages OR sugar-sweetened beverage OR sugar-sweetened beverages OR ((ssb[tiab] OR ssbs[tiab]) AND (beverage*[tiab] OR drink*[tiab] OR juic*[tiab] OR soda*[tiab])) OR sweet drink OR sweet drinks OR sweet beverage OR sweet beverages OR sweetened drink OR sweetened drinks OR sweetened beverage OR sweetened beverages OR sugary drink OR sugary drinks OR sugary beverage OR sugary beverages OR carbonated beverage OR carbonated beverages OR carbonated drink OR carbonated drinks OR soft drink OR soft drinks OR soda[tiab] OR sodas[tiab] OR soda-pop OR soda pop OR soda pops OR cola[tiab] OR coca-cola OR flavored water OR flavored waters OR flavoured water OR flavoured waters OR juice OR juices OR fruit-flavored drink OR fruit-flavored drinks OR fruit-flavoured drink OR fruit-flavoured drinks OR fruit-flavored beverage OR fruit-flavored beverages OR fruit-flavoured beverage OR fruit-flavoured beverages OR drink[tiab] OR drinks[tiab] OR beverage[tiab] OR beverages[tiab] OR carbonated beverages[mesh]) AND (warning*[tiab] OR label*[tiab] OR fop[tiab] OR front-of-package OR “front of package” OR “Traffic light” OR tls[tiab] OR tll[tiab] OR "health claim" OR "health claims" OR messag*[tiab] OR ((product labeling[mesh] OR food labeling[mesh]) AND warning*[tiab])) NOT (letter[pt] OR comment[pt] OR news[pt]) | 3,312 | 106 |
| Scopus | TITLE-ABS-KEY ( "sugar sweetened beverage" OR "sugar sweetened beverages" OR "sugar-sweetened beverage" OR "sugar-sweetened beverages" OR "ssb" OR "ssbs" OR "sweet drink" OR "sweet drinks" OR "sweet beverage" OR "sweet beverages" OR "sweetened drink" OR "sweetened drinks" OR "sweetened beverage" OR "sweetened beverages" OR "sugary drink" OR "sugary drinks" OR "sugary beverage" OR "sugary beverages" OR "carbonated beverage" OR "carbonated beverages" OR "carbonated drink" OR "carbonated drinks" OR "soft drink" OR "soft drinks" OR "soda" OR "sodas" OR "soda-pop" OR "soda pop" OR "soda pops" OR "cola" OR "coca-cola" OR "flavored water" OR "flavored waters" OR "flavoured water" OR "flavoured waters" OR "juice" OR "juices" OR "fruit-flavored drink" OR "fruit-flavored drinks" OR "fruit-flavoured drink" OR "fruit-flavoured drinks" OR "fruit-flavored beverage" OR "fruit-flavored beverages" OR "fruit-flavoured beverage" OR "fruit-flavoured beverages" OR drink OR drinks OR "beverage" OR "beverages" OR "carbonated beverage*" ) AND TITLE-ABS-KEY ( ( warning AND ( label OR labels OR labelling OR labellings ) ) OR fop OR "traffic light" OR tls OR tll OR "front-of-package" OR "front of package" OR "front of pack" OR "front-of-pack" OR "health claim" OR "health claims" OR messag* OR "product label*" ) | 1,499 | 143 |
| Cochrane Central | (("sugar sweetened beverage" OR "sugar sweetened beverages" OR "sugar-sweetened beverage" OR "sugar-sweetened beverages" OR ((ssb OR ssbs) AND (beverage* OR drink* OR juic* OR soda*)) OR "sweet drink" OR "sweet drinks" OR "sweet beverage" OR "sweet beverages" OR "sweetened drink" OR "sweetened drinks" OR "sweetened beverage" OR "sweetened beverages" OR "sugary drink" OR "sugary drinks" OR "sugary beverage" OR "sugary beverages" OR "carbonated beverage" OR "carbonated beverages" OR "carbonated drink" OR "carbonated drinks" OR "soft drink" OR "soft drinks" OR soda OR sodas OR soda-pop OR "soda pop" OR "soda pops" OR cola OR coca-cola OR "flavored water" OR "flavored waters" OR "flavoured water" OR "flavoured waters" OR juice OR juices OR "fruit-flavored drink" OR "fruit-flavored drinks" OR "fruit-flavoured drink" OR "fruit-flavoured drinks" OR "fruit-flavored beverage" OR "fruit-flavored beverages" OR "fruit-flavoured beverage" OR "fruit-flavoured beverages" OR drink OR drinks OR beverage OR beverages OR [mh "carbonated beverages"]) AND (warning* OR label* OR fop OR front-of-package OR "front of package" OR "Traffic light" OR tls OR tll OR "health claim" OR "health claims" OR messag* OR (([mh "product labeling"] OR [mh "food labeling"]) AND warning*))):ti,ab,kw" | 8 | 1 |
| Embase.com | ('sugar-sweetened beverage'/exp OR 'sugar sweetened beverage' OR 'sugar sweetened beverages' OR 'sugar-sweetened beverage' OR 'sugar-sweetened beverages' OR ((ssb:ti,ab OR ssbs:ti,ab) AND (beverage*:ti,ab OR drink*:ti OR juic*:ti,ab OR soda*:ti,ab)) OR 'sweet drink' OR 'sweet drinks' OR 'sweet beverage' OR 'sweet beverages' OR 'sweetened drink' OR 'sweetened drinks' OR 'sweetened beverage' OR 'sweetened beverages' OR 'sugary drink' OR 'sugary drinks' OR 'sugary beverage' OR 'sugary beverages' OR 'carbonated beverage' OR 'carbonated beverages' OR 'carbonated drink' OR 'carbonated drinks' OR 'soft drink' OR 'soft drinks' OR soda:ti,ab OR sodas:ti,ab OR 'soda pop' OR 'soda pops' OR cola:ti,ab OR 'coca cola' OR 'flavored water' OR 'flavored waters' OR 'flavoured water' OR 'flavoured waters' OR juice OR juices OR 'fruit-flavored drink' OR 'fruit-flavored drinks' OR 'fruit-flavoured drink' OR 'fruit-flavoured drinks' OR 'fruit-flavored beverage' OR 'fruit-flavored beverages' OR 'fruit-flavoured beverage' OR 'fruit-flavoured beverages' OR drink:ti,ab OR drinks:ti,ab OR beverage:ti,ab OR beverages:ti,ab OR 'carbonated beverages'/exp) AND (warning*:ti,ab OR label*:ti,ab OR fop:ti,ab OR 'front of package' OR 'traffic light' OR tls:ti,ab OR tll:ti,ab OR 'health claim' OR 'health claims' OR messag*:ti,ab OR (('labeling'/exp OR 'food packaging'/exp) AND warning*:ti,ab)) AND (‘article’/it OR ‘article in press’/it OR ‘review’/it) | 2,106 | 217 |
| CINAHL via EBSCOhost | ( ((MH "Carbonated Beverages") OR (MH "Energy Drinks") OR (MH "Fruit Juices+") OR (MH "Carbonated Beverages") OR (MH "Energy Drinks") OR (MH "Fruit Juices+") OR (MH Sweetened Beverages") OR (MH "Sports Drinks") OR "sugar sweetened beverage" OR "sugar sweetened beverages" OR "sugar-sweetened beverage" OR "sugar-sweetened beverages" OR TI ssb OR AB ssb OR TI ssbs OR AB ssbs OR TI beverage* OR AB beverage* OR TI drink* OR TI juic* OR AB juic* OR TI soda* OR AB soda* OR "sweet drink" OR "sweet drinks" OR "sweet beverage" OR "sweet beverages" OR "sweetened drink" OR "sweetened drinks" OR "sweetened beverage" OR "sweetened beverages" OR "sugary drink" OR "sugary drinks" OR "sugary beverage" OR "sugary beverages" OR "carbonated beverage" OR "carbonated beverages" OR "carbonated drink" OR "carbonated drinks" OR "soft drink" OR "soft drinks" OR TI soda OR AB soda OR TI sodas OR AB sodas OR soda-pop OR "soda pop" OR "soda pops" OR TI cola OR AB cola OR coca-cola OR "flavored water" OR "flavored waters" OR "flavoured water" OR "flavoured waters" OR juice OR juices OR "fruit-flavored drink" OR "fruit-flavored drinks" OR "fruit-flavoured drink" OR "fruit-flavoured drinks" OR "fruit-flavored beverage" OR "fruit-flavored beverages" OR "fruit-flavoured beverage" OR "fruit-flavoured beverages" OR TI drink OR AB drink OR TI drinks OR AB drinks OR TI beverage OR AB beverage OR TI beverages OR AB beverages) ) AND ( (TI warning* OR AB warning* OR TI label* OR AB label* OR TI fop OR AB fop OR front-of-package OR "front of package" OR "Traffic light" OR TI tls OR AB tls OR TI tll OR AB tll OR "health claim" OR "health claims" OR TI messag* OR AB messag* OR ((MH "product labeling+") OR (MH "food labeling+") AND (TI warning* OR AB warning*)) ) Limit to academic journals | 636 | 148 |
| PsycINFO via EBSCOhost | ( ((DE "Beverages (Nonalcoholic)" OR DE "Energy Drink") OR "sugar sweetened beverage" OR "sugar sweetened beverages" OR "sugar-sweetened beverage" OR "sugar-sweetened beverages" OR TI ssb OR AB ssb OR TI ssbs OR AB ssbs OR TI beverage* OR AB beverage* OR TI drink* OR TI juic* OR AB juic* OR TI soda* OR AB soda* OR "sweet drink" OR "sweet drinks" OR "sweet beverage" OR "sweet beverages" OR "sweetened drink" OR "sweetened drinks" OR "sweetened beverage" OR "sweetened beverages" OR "sugary drink" OR "sugary drinks" OR "sugary beverage" OR "sugary beverages" OR "carbonated beverage" OR "carbonated beverages" OR "carbonated drink" OR "carbonated drinks" OR "soft drink" OR "soft drinks" OR TI soda OR AB soda OR TI sodas OR AB sodas OR soda-pop OR "soda pop" OR "soda pops" OR TI cola OR AB cola OR coca-cola OR "flavored water" OR "flavored waters" OR "flavoured water" OR "flavoured waters" OR juice OR juices OR "fruit-flavored drink" OR "fruit-flavored drinks" OR "fruit-flavoured drink" OR "fruit-flavoured drinks" OR "fruit-flavored beverage" OR "fruit-flavored beverages" OR "fruit-flavoured beverage" OR "fruit-flavoured beverages" OR TI drink OR AB drink OR TI drinks OR AB drinks OR TI beverage OR AB beverage OR TI beverages OR AB beverages) ) AND ( (DE "Warning Labels") OR (DE "food labelling") OR TI warning* OR AB warning* OR TI label* OR AB label* OR TI fop OR AB fop OR front-of-package OR "front of package" OR "Traffic light" OR TI tls OR AB tls OR TI tll OR AB tll OR "health claim" OR "health claims" OR TI messag* OR AB messag*) ) limit to academic journals | 1,003 | 41 |
| Communication and Mass Media Complete via EBSCOhost | ("sugar sweetened beverage" OR "sugar sweetened beverages" OR "sugar-sweetened beverage" OR "sugar-sweetened beverages" OR ((ssb OR ssbs) AND (beverage* OR drink* OR juic* OR soda*)) OR "sweet drink" OR "sweet drinks" OR "sweet beverage" OR "sweet beverages" OR "sweetened drink" OR "sweetened drinks" OR "sweetened beverage" OR "sweetened beverages" OR "sugary drink" OR "sugary drinks" OR "sugary beverage" OR "sugary beverages" OR "carbonated beverage" OR "carbonated beverages" OR "carbonated drink" OR "carbonated drinks" OR "soft drink" OR "soft drinks" OR soda OR sodas OR soda-pop OR "soda pop" OR "soda pops" OR cola OR coca-cola OR "flavored water" OR "flavored waters" OR "flavoured water" OR "flavoured waters" OR juice OR juices OR "fruit-flavored drink" OR "fruit-flavored drinks" OR "fruit-flavoured drink" OR "fruit-flavoured drinks" OR "fruit-flavored beverage" OR "fruit-flavored beverages" OR "fruit-flavoured beverage" OR "fruit-flavoured beverages" OR drink OR drinks OR beverage OR beverages OR "carbonated beverages") AND (warning* OR label* OR fop OR front-of-package OR "front of package" OR "Traffic light" OR tls OR tll OR "health claim" OR "health claims" OR messag* OR (("product labeling" OR "food labeling") AND warning*)) limit to “academic journals, trade publications and reviews | 410 | 4 |
| **Total records before removing duplicates** | | 8,974 | 660 |
