# Supplementary material for: Sugary drink warnings: A meta-analysis of experimental studies
Source: PLoS Med. 2020 May 20;17(5):e1003120. doi: 10.1371/journal.pmed.1003120 (PMC7239392; doi:10.1371/journal.pmed.1003120)
Supplement: S3 Table — (DOCX) [file pmed.1003120.s019.docx]

**S3 Table.** Definitions of relevant interventions and comparators. To be eligible, studies must report results for at least one relevant intervention and at least one relevant comparator.

| **Condition** | **Definition** | **Example** |
| --- | --- | --- |
| **Interventions** |  |  |
| ***Relevant warning topics*** | | |
| Health warning | A direct statement about health effects of consuming a nutrient or product.   - May specify a referent to whom the harms apply (e.g., “soda contributes to obesity [in children]”). - May directly reference consumption or may only reference the product (e.g., “Drinking soda causes” vs. “Soda causes” are both acceptable). - May contain a signal/marker word such as “WARNING” or “SAFETY WARNING.” - May be text-only or may include a photograph or icon. | *State of California Warning: Drinking beverages with added sugar(s) contributes to obesity, diabetes, and tooth decay.* |
| Nutrient warning | A message the alerts consumers that a product has a high amount of a harmful nutrient (i.e., sugar, salt, fat, saturated fat, trans fat, or calories).   - Must contain either a signal/marker word (“WARNING”) or a “High in” statement (or the equivalent in another language) that signals high or unhealthy levels of a harmful nutrient. “High In” statements include: “High in,” “Excess of,” or “High.” In Spanish, “High In” statements include “Alto en,” “Exceso de,” and “Alto.” Statements without a marker word and without a High In statement are not considered warnings and are excluded from “warnings.” See below regarding these statements as comparators. - May include information on exact nutrient content (e.g., number of calories or number of grams of sugar). - May be text-only or may include a photograph or icon. | *WARNING: High in sugar.* |
| ***Relevant warning types*** | | |
| Text-only warning | A health or nutrient warning that does not include any images. | *Warning: Excess sugar intake causes dental decay.* |
| Icon pictorial warnings | A health or nutrient warning that includes an icon (e.g., a hazard symbol, a symbol depicting the warning message such as an icon of a diseased heart or an icon of a spoonful of sugar).   - Must contain a text health or nutrient warning. Icons only without accompanying warning text are NOT eligible. - Icon may or may not “match” the text (e.g., may depict the health effect described, or not; may depict the nutrient described, or not). | 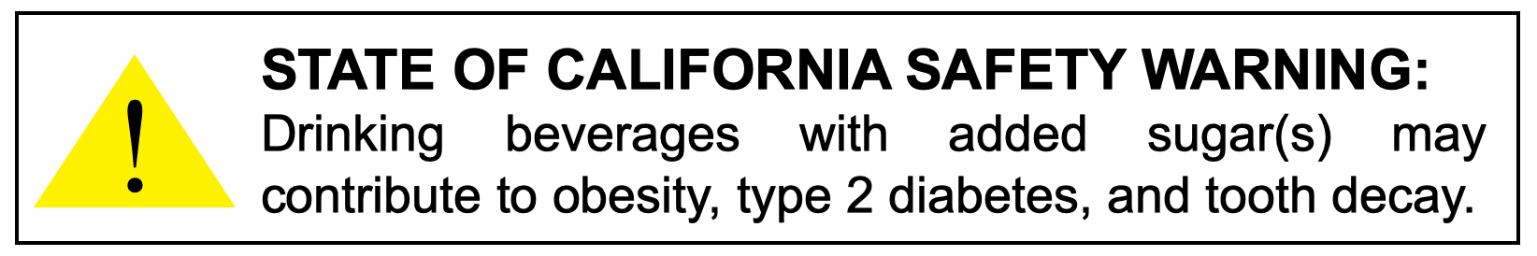 |
| Graphic pictorial warning | A health or nutrient warning that includes a photograph.   - Must contain a text health or nutrient warning. Photographs only without accompanying warning text are NOT eligible. - Image may or may not “match” the text (e.g., may depict the health effect described, or not; may depict the nutrient described, or not). | 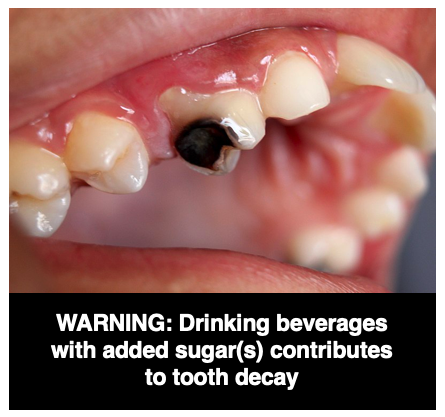  Image source: <https://commons.wikimedia.org/wiki/File:Dental_Caries_Cavity_2.JPG> |
| **Comparators** |  |  |
| No label | No label is shown in the control condition. | Image of the product is displayed without a label. |
| Neutral image or message | Control label depicts a neutral image (e.g., a barcode) or a neutral message (e.g., a message about littering, or a prompt to read the Nutrition Facts Panel). | *This product is not biodegradable. Always recycle.* |
| Calorie content label | Control label indicates calorie content of the product, but does NOT include a marker word such as “WARNING” **and** does NOT include a “High In” statement.   - May specify calorie content per serving or per product (e.g. “120 calories per serving” and “240 calories per bottle” are both acceptable). - May specify % daily value (e.g., 10% DV). | 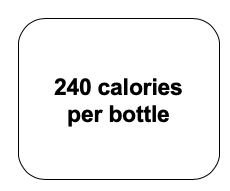 |
| Nutrient content label | Control label indicates sugar, salt, fat, or other nutrient content of the product but does NOT include a marker word such as “WARNING” and does NOT include a “High in” or other interpretative statement. Does NOT include any color coding to communicate healthfulness. This includes Facts Up Front and Daily Guideline Amount labels, as long as they are not color-coded in a traffic light scheme. Does NOT include labels with photographs.   - May include one or more nutrients, for example, only sugar content, or sugar, calories, and salt. - May specify that nutrient content is per serving or per product. - May specify percent daily value (e.g., 10% DV). - May NOT use color coding to indicate high or low amounts of nutrients. For example, traffic light labels are NOT eligible comparators. | 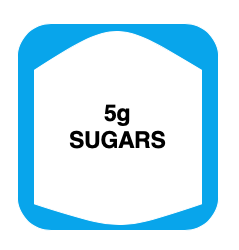 |
