# Supplementary material for: Sugary drink warnings: A meta-analysis of experimental studies
Source: PLoS Med. 2020 May 20;17(5):e1003120. doi: 10.1371/journal.pmed.1003120 (PMC7239392; doi:10.1371/journal.pmed.1003120)
Supplement: S4 Table — (DOCX) [file pmed.1003120.s020.docx]

**S4 Table.** Effectiveness of sugary drink warnings vs. control: mean weighted effect sizes (*d* with Hedges’s correction) and heterogeneity statistics (*k*=23 studies), sensitivity analysis.

| **Outcome** | **N** | **k** | ***d*** | **95% CI** | | **Q** | **Qp** | **I^2^** |
| --- | --- | --- | --- | --- | --- | --- | --- | --- |
| **Behavior** |  |  |  |  |  |  |  |  |
| Purchases of sugary drinks (*primary outcome) | 1,407 | 4 | **-.16** | **(-.30** | **, -.03)** | 3.93 | 0.269 | 12.77 |
| Calories purchased from beverages | 2,338 | 3 | **-.16** | **(-.24** | **, -.07)** | 1.23 | 0.540 | .01 |
| Grams of sugar purchased from beverages | 1,938 | 2 | **-.11** | **(-.21** | **, -.01)** | .00 | 0.949 | .00 |
| **Attention and noticing** |  |  |  |  | |  |  |  |
| Noticed nutrition or trial label | 1,840 | 2 | **.83** | **(.54** | **, 1.12)** | 6.36 | 0.012 | 84.29 |
| **Warning reactions** |  |  |  |  |  |  |  |  |
| Negative emotional reactions | 3,594 | 4 | **.69** | **(.25** | **, 1.13)** | 137.52 | <0.001 | 97.39 |
| Thinking about the health effects of sugary drinks | 2,543 | 4 | **.65** | **(.29** | **, 1.01)** | 41.54 | <0.001 | 93.69 |
| **Attitudes and beliefs about sugary drinks** |  |  |  |  |  |  |  |  |
| Healthfulness perceptions | 6,947 | 9 | **-.22** | **(-.27** | **, -.17)** | 8.15 | 0.419 | 1.68 |
| Positive outcome expectancies | 4,583 | 2 | **-.26** | **(-.34** | **, -.17)** | 3.14 | 0.076 | 68.19 |
| Positive product attitudes | 5,969 | 6 | -.54 | (-1.44 | , .35) | 212.18 | <0.001 | 99.66 |
| Perceived disease likelihood | 7,072 | 6 | **.15** | **(.06** | **, .24)** | 26.51 | <0.001 | 76.46 |
| Amount of added sugar | 4,983 | 3 | .25 | (-.05 | , .55) | 38.55 | <0.001 | 95.37 |
| **Policy support** | 2,132 | 2 | .19 | (-.14 | , .51) | 13.86 | <0.001 | 92.79 |
| **Intentions and hypothetical choices** |  |  |  |  |  |  |  |  |
| Hypothetical purchases of sugary drinks | 7,681 | 6 | **-.33** | **(-.44** | **, -.21)** | 14.42 | 0.013 | 77.93 |
| Purchase or consumption intentions | 7,118 | 8 | **-.30** | **(-.44** | **, -.15)** | 38.61 | <0.001 | 88.53 |
| Hypothetical coupon selection, sugary drinks | 4,583 | 2 | **-.31** | **(-.37** | **, -.25)** | .00 | 0.972 | .04 |
| Hypothetical coupon selection, non-sugary drinks | 4,583 | 2 | -.02 | (-.21 | , .17) | 9.57 | 0.002 | 89.55 |
| Hypothetical total expenditure on beverages | 1,189 | 2 | -.08 | (-.21 | , .06) | .46 | 0.495 | .00 |

*Note.* N, number of participants; k, number of effect sizes; *d*, corrected standardized mean difference (pooled effect size). **Bold** effect sizes are statistically significant at *p*< .05. Sensitivity analyses assumed a correlation 0.5 among combined similar dependent variables (vs. 0.0 correlation in primary analyses).
