# Supplementary material for: Sugary drink warnings: A meta-analysis of experimental studies
Source: PLoS Med. 2020 May 20;17(5):e1003120. doi: 10.1371/journal.pmed.1003120 (PMC7239392; doi:10.1371/journal.pmed.1003120)
Supplement: S5 Table — (DOCX) [file pmed.1003120.s021.docx]

**S5 Table.** Studies included in the review.

| **Study** | **Country & Population** | **Study design^a^** | **Warning topic(s) examined** | **Warning type(s) examined** | **Comparator(s) examined** | **Warning Setting** | **Exposure medium** | **Dependent variable(s) assessed^b^** | **Included in meta-analysis?** |
| --- | --- | --- | --- | --- | --- | --- | --- | --- | --- |
| Acton (2018)^1^ | Canada adolescent and adult shoppers (ages 16+) | Between-subjects | - Nutrient - Health | Text | No label (image of product) | Computer survey | Warning on front-of-package | - % selecting a sugary drink (real-stakes) - Free sugar purchased from beverages (real-stakes) - Sodium purchased from beverages (real-stakes) - Saturated fat purchased from beverages (real-stakes) - Calories purchased from beverages (real-stakes) | Yes |
| Acton (2019)^2^ | Canada adolescents and adults (ages 13+) | Between-subjects | Nutrient | Pictorial – Icon | No label (image of product) | Paper survey (large posterboard) | Warning on front-of-package | - % noticing nutrition labels or symbols - Free sugar purchased from beverages (real-stakes) - Calories purchased from beverages (real-stakes) | Yes |
| Ang (2019)^3^ | Singapore adults (ages 21+) | Between-subjects | - Nutrient - Health | Text | No label (image of product) | Naturalistic online store | Warning shown next to image of product | - Proportion of high-in sugar beverages selected (hypothetical) - Total sugar purchased from beverages (hypothetical) - Sugar purchased per dollar spent on beverages (hypothetical) - Total dollar spent on beverages(hypothetical) - Total expenditure on high-in-sugar beverages (hypothetical) | Yes |
| Ares (2018)^4^ Study 1 | Uruguay adults | Within-subjects | Nutrient | Text | No label (image of product) | Computer survey | Warning on front-of-package | - Attentional capture of labels (response time to identify presence of label) | No |
| Ares (2018)^4^ Study 2 | Uruguay adults | Within-subjects | Nutrient | Text | No label (image of product) | Computer survey | Warning on front-of-package | - Time required to evaluate product healthfulness - Classification of products as healthful | No |
| Ares (2018)^4^ Study 3 | Uruguay adults | Between-subjects | Nutrient | Text | No label (image of product) | Computer survey | Warning on front-of-package | - Purchase intention - Perceived healthfulness | Yes |
| Arrua (2017)^5^ | Uruguay children (grades 4-6) | Within-subjects | Nutrient | Text | No label (image of product) | Paper survey | Warning on front-of-package | - Choice of product in discrete choice experiment (hypothetical) | No |
| Billich (2018)^6^ | Australia young adults (ages 18-35) | Between-subjects | Health | - Text - Pictorial-graphic | No label (image of product) | Computer survey | Warning shown next to image of product | - % selecting an SSB in choice scenario (hypothetical) - % selecting a diet drink in choice scenario (hypothetical) - % selecting a high Health Star Rating drink in choice scenario (hypothetical) - % of participants indicating they considered healthiness of drink selected in choice scenario - Perception of impact of drinking SSBs has on health | Yes |
| Bollard (2016)^7^ | New Zealand adolescent and adult (ages 13-24) sugary drink consumers | Between-subjects | - Health - Nutrient | - Text - Pictorial-graphic | No label (image of product) | Computer survey | Warning on front-of-package | - Product attractiveness - Product taste - Product quality - Product coolness - Product healthfulness - Product cost - Consumer interest - Consumer fashionableness - Consumer age - Likelihood to buy | Yes |
| David (2018)^8^ | Brazil university students | Within-subjects | Health + nutrient | Text | Neutral message (“This product contains sugar and must be kept under refrigeration and be consumed before the expiration date”) | Projected on a screen | Warning shown before image of product | - Appetitive drive - Arousal - Valence - Intention to consume | Yes |
| Donnelly (2018)^9^ Study 1 | United States sugary drink consumers | Between-subjects | Health | Pictorial - graphic | No label (image of logo for preferred soda brand) | Computer survey | Warning next to image of product | - Negative affect - Health consideration - Intention to purchase water vs. soda | Yes |
| Donnelly (2018)^9^ Study 2 | United States adults (nationally representative sample) | Within-subjects | Health | - Text - Pictorial-graphic | Calorie content label | Computer survey | Warning by itself | - Consumer support | Yes |
| Grummon (2019a)^10^ | United States adults | Between subjects | - Health - Nutrient - Health + nutrient | Text | Neutral message (“Always read the Nutrition Facts Panel”) | Computer survey | Warning on front-of-package | - Perceived message effectiveness - Fear - Thinking about harms | Yes |
| Grummon (2019b)^11^ | United States adult sugary drink consumers | Between subjects | Health | Text | Neutral label (image of a barcode) | Naturalistic laboratory store | Warning on front-of-package | - Calories purchased from SSBs - Calories purchased from non-SSBs - Calories purchased from Foods - Total calories purchased - Purchase of an SSB (%) - Number of SSBs purchased - Intentions to limit consumption of beverages with added sugar - Intentions to limit consumption of SSBs in trial store - Noticed trial label (%) - Attention to label - Thinking about warning message/harms - Negative emotions elicited by label - Anticipated social interactions about label - Perceived amount of added sugar - Perceived healthfulness - Positive product attitudes - Negative outcome expectations | Yes |
| Hayward (2019)^12^ Study 1 | United States participants | Between-subjects | Health | Graphic – Pictorial | No label (image of logo for preferred soda brand) | Computer survey | Warning next to image of product | - Purchase intentions (hypothetical) - Disgust - Universal Measure of Bias (total) - Negative judgment - Social distance - Unattractiveness - Denial of equal rights | Yes |
| Hayward (2019)^12^, Study 2 | United States participants who self-identified as overweight or obese | Between-subjects | Health | Graphic – Pictorial | No label (participants completed measures before exposure to any images or messages) | Computer survey | Warning by itself | - Perceived personal stigma - Perceived obesity stigma - Negative mood - Positive mood - State self-esteem (overall) - Appearance self-esteem | Yes |
| Lima (2018)^13^, Parents | Brazil parents of children ages 6-12 | Between-subjects | Nutrient | Text | Nutrient content label (Guideline Daily Amount) | Computer survey | Warning on front-of-package | - Healthfulness perceptions - Ideal consumption frequency | Yes |
| Lima (2018)^13^, Children | Brazil children ages 6-12 | Between-subjects | Nutrient | Text | Nutrient content label (Guideline Daily Amount) | Computer survey | Warning on front-of-package | - Perceived healthfulness | Yes |
| Machín (2018)^14^ | Uruguay adults | Between subjects | Nutrient | Text | ­No label (image of product) | Computer survey | Warning next to image or product | - Expenditure on beverages (hypothetical) - Expenditure on juice (hypothetical) | Yes |
| Mantzari (2018)^15^ | United Kingdom parents of children ages 11-16 in households consuming sugary drinks | Between-subjects | Health | Graphic – Pictorial | - No label (image of product) - Calorie content label | Computer survey | Warning on front-of-package | - % choosing SSB in vending machine task (hypothetical) - Negative emotional arousal - Perceived health risks (aggregate) - Weight gain - Develop heart disease - Develop diabetes - Lead healthy life - Acceptability | Yes |
| Mantzari (2019)^16^ | United Kingdom adults | Between-subjects | Health | Graphic – Pictorial | - No label (unaltered product) - Calorie content label | Naturalistic laboratory pantry | Warning on front-of-package | - % choosing an SSB (real-stakes) | Yes |
| Nobrega (2020)^17^ | Brazil adults | Within-subjects | Nutrient | Text | No label (image of product) | Computer survey | Warning on front-of-package | - Perceived healthfulness | Yes |
| Popova (2019)^18^ | United States young adults (ages 18-34) | Between-subjects | Health | Text | No label (image of product) | Computer survey | Warning on front-of-package | - Attention to warning – Fixation count - Attention to warning – Dwell time - Correct recall of warning message - Correct recall of “Clean, crisp, refreshing” - Correct recall of “Cola” - Correct recall of size - Product appeal – Cheap – Expensive - Product appeal – Unattractive – Attractive - Product appeal – Low – High Quality - Product appeal – Unhealthy – Healthy - Product appeal – Tasted Bad – Good - Product appeal – Average score - Risk perceptions – Weight gain - Risk perceptions – Heart disease - Risk perceptions – Diabetes - Risk perceptions – Tooth decay - Risk perceptions – Average score - Purchase intentions | Yes |
| Roberto (2016)^19^ | United States primary caregivers of children ages 6-11 | Between-subjects | Health | Text | - No label (image of product) - Calorie content label | Computer survey | Warning on front-of-package | - % choosing an SSB in vending machine choice task (hypothetical) - SSB perceptions – Child would find delicious - SSB perceptions – Healthy - Purchase intentions - Willingness to pay - Allow child to drink - SSB perceptions – Make child feel energized - SSB perceptions – Help child focus - SSB perceptions – Amount of added sugar - SSB perceptions – Estimated calories - SSB disease risk – Weight gain - SSB disease risk – Heart disease - SSB disease risk – Diabetes - SSB disease risk – Healthy life - Coupon choice – Number of SSB coupons (hypothetical) - Coupon choice – Number of non-SSB coupons (hypothetical) | Yes |
| Temple (2016)^20^ | United States adolescents and adults (ages 15-30) | Within-subjects | Health | Text | - No label (unaltered product) - Nutrient content label (milligrams of caffeine) | Naturalistic laboratory store | Warning on front-of-package | - Servings of energy drinks purchased | Yes |
| VanEpps (2016)^21^ | United States adolescents (ages 12-18) | Between-subjects | Health | Text | - No label (image of product) - Calorie content label | Computer survey | Warning on front-of-package | - % choosing an SSB in vending machine choice task (hypothetical) - SSB perceptions – Delicious - SSB perceptions – Healthy - Purchase intentions - SSB perceptions - Energized - SSB perceptions – Focus - SSB perceptions – Amount of added sugar - SSB perceptions – Estimated calories - SSB disease risk – Weight gain - SSB disease risk – Heart disease - SSB disease risk – Diabetes - SSB disease risk – Healthy life - Coupon choice – Number of SSB coupons (hypothetical) - Coupon choice – Number of non-SSB coupons (hypothetical) | Yes |

Abbreviations: SSB, sugar-sweetened beverage

^a^Refers to type of randomization used for relevant warning vs. control comparison(s).

^b^All dependent variables relevant to sugary drink warnings are listed, including outcomes that were not meta-analyzable. Variables are listed as authors defined them.

**References**

1. Acton R, Hammond D. The impact of price and nutrition labelling on sugary drink purchases: Results from an experimental marketplace study. *Appetite*. 2018;121:129-137.

2. Acton R, Jones A, Kirkpatrick S, Roberto C, Hammond D. Taxes and front-of-package labels improve the healthiness of beverage and snack purchases: A randomized experimental marketplace. *International Journal of Behavioral Nutrition and Physical Activity*. 2019;16(1):46. doi:10.1186/s12966-019-0799-0

3. Ang FJL, Agrawal S, Finkelstein EA. Pilot randomized controlled trial testing the influence of front-of-pack sugar warning labels on food demand. *BMC Public Health*. 2019;19(1):164. doi:10.1186/s12889-019-6496-8

4. Ares G, Varela F, Machin L, et al. Comparative performance of three interpretative front-of-pack nutrition labelling schemes: Insights for policy making. *Food Quality and Preference*. 2018;68:215-225.

5. Arrua A, Curutchet MR, Rey N, et al. Impact of front-of-pack nutrition information and label design on children’s choice of two snack foods: Comparison of warnings and the traffic-light system. *Appetite*. 2017;116:139-146. doi:10.1016/j.appet.2017.04.012

6. Billich N, Blake MR, Backholer K, Cobcroft M, Li V, Peeters A. The effect of sugar-sweetened beverage front-of-pack labels on drink selection, health knowledge and awareness: An online randomised controlled trial. *Appetite*. 2018;128:233-241. doi:10.1016/j.appet.2018.05.149

7. Bollard T, Maubach N, Walker N, Mhurchu CN. Effects of plain packaging, warning labels, and taxes on young people’s predicted sugar-sweetened beverage preferences: An experimental study. *Int J Behav Nutr Phys Act*. 2016;13(1):95.

8. David IA, Krutman L, Fernandez-Santaella MC, et al. Appetitive drives for ultra-processed food products and the ability of text warnings to counteract consumption predispositions. *Public Health Nutr*. 2018;21(3):543-557. doi:10.1017/s1368980017003263

9. Donnelly G, Zatz L, Svirsky D, John L. The effect of graphic warnings on sugary-drink purchasing. *Psych Science*. 2018;29(8):1321-1333.

10. Grummon AH, Hall MG, Taillie LS, Brewer NT. How should sugar-sweetened beverage health warnings be designed? A randomized experiment. *Prev Med*. 2019a;121:158-166. doi:10.1016/j.ypmed.2019.02.010

11. Grummon AH, Taillie LS, Golden SD, Hall MG, Ranney LM, Brewer NT. Sugar-sweetened beverage health warnings and purchases: A randomized controlled trial. *Am J Prev Med*. 2019b. doi:https://doi.org/10.1016/j.amepre.2019.06.019

12. Hayward L, Vartanian L. Potential unintended consequences of graphic warning labels on sugary drinks: Do they promote obesity stigma? *Obesity Science & Practice*.

13. Lima M, Ares G, Deliza R. How do front of pack nutrition labels affect healthfulness perception of foods targeted at children? Insights from Brazilian children and parents. *Food Quality and Preference*. 2018;64:111-119. doi:10.1016/j.foodqual.2017.10.003

14. Machín L, Aschemann-Witzel J, Curutchet MR, Giménez A, Ares G. Does front-of-pack nutrition information improve consumer ability to make healthful choices? Performance of warnings and the traffic light system in a simulated shopping experiment. *Appetite*. 2018;121:55-62. doi:10.1016/j.appet.2017.10.037

15. Mantzari E, Vasiljevic M, Turney I, Pilling M, Marteau T. Impact of warning labels on sugar-sweetened beverages on parental selection: An online experimental study. *Preventive Medicine Reports*. 2018;12:259-267. doi:10.1016/j.pmedr.2018.10.016

16. Mantzari E, Pechey R, Codling S, Sexton O, Hollands GJ, Marteau TM. The impact of ‘on-pack’pictorial health warning labels and calorie information labels on drink choice: A laboratory experiment. *Appetite*. 2019:104484.

17. Nobrega L, Ares G, Deliza R. Are nutritional warnings more efficient than claims in shaping consumers’ healthfulness perception? *Food Quality and Preference*. 2020;79. doi:10.1016/j.foodqual.2019.103749

18. Popova L, Nonnemaker J, Taylor N, Bradfield B, Kim A. Warning labels on sugar-sweetened beverages: An eye tracking approach. *Am J Health Behav*. 2019;43(2):406-419.

19. Roberto CA, Wong D, Musicus A, Hammond D. The Influence of Sugar-Sweetened Beverage Health Warning Labels on Parents’ Choices. *Pediatrics*. 2016;137(2):e20153185. doi:10.1542/peds.2015-3185

20. Temple JL, Ziegler AM, Epstein LH. Influence of Price and Labeling on Energy Drink Purchasing in an Experimental Convenience Store. *J Nutr Educ Behav*. 2016;48(1):54-59.e1. doi:10.1016/j.jneb.2015.08.007

21. VanEpps EM, Roberto CA. The Influence of Sugar-Sweetened Beverage Warnings: A Randomized Trial of Adolescents’ Choices and Beliefs. *Am J Prev Med*. 2016;51(5):664-672. doi:10.1016/j.amepre.2016.07.010
