# Supplementary material for: Sugary drink warnings: A meta-analysis of experimental studies
Source: PLoS Med. 2020 May 20;17(5):e1003120. doi: 10.1371/journal.pmed.1003120 (PMC7239392; doi:10.1371/journal.pmed.1003120)
Supplement: S6 Table — (DOCX) [file pmed.1003120.s022.docx]

**S6 Table.** Moderation of effects of sugary drink warnings vs. control.

|  | **N** | **k** | ***d*** | **95% CI** | | ***Q*_b_** | **p** |
| --- | --- | --- | --- | --- | --- | --- | --- |
| **Hypothetical selection or purchase of sugary drinks** |  |  |  |  |  |  |  |
| Warning topic |  |  |  |  |  |  |  |
| Health warning | 6,707 | 5 | **-.35** | **(-0.47** | **, -0.24)** | **4.04** | **0.044** |
| Nutrient warning | 974 | 2 | **-.18** | **(-0.31** | **, -0.05)** |  |  |
| **Purchase or consumption intentions** |  |  |  |  |  |  |  |
| Sugary drink consumption status of sample |  |  |  |  |  |  |  |
| All sugary drink consumers | 1,206 | 3 | **-.40** | **(-0.77** | **, -0.02)** | .54 | 0.463 |
| Mixed consumers & non-consumers or not reported | 5,912 | 5 | **-.25** | **(-0.38** | **, -0.12)** |  |  |
| Ages included in sample |  |  |  |  |  |  |  |
| Included children | 2,806 | 2 | **-.16** | **(-0.29** | **, -0.03)** | 2.49 | 0.115 |
| Did not include children | 4,312 | 6 | **-.34** | **(-0.53** | **, -0.16)** |  |  |
| **Perceived disease likelihood** |  |  |  |  |  |  |  |
| Sugary drink consumption status of sample |  |  |  |  |  |  |  |
| All sugary drink consumers | 1,728 | 2 | **.13** | **(0.03** | **, 0.22)** | .10 | 0.755 |
| Mixed consumers & non-consumers or not reported | 5,344 | 4 | **.15** | **(0.01** | **, 0.30)** |  |  |

*Note.* N, number of participants; k, number of studies or effect sizes; *d*, corrected standardized mean difference (pooled effect size). **Bold** effect sizes and *Q*_b_ statistics are statistically significant at *p*< .05.
