# Supplementary material for: Sugary drink warnings: A meta-analysis of experimental studies
Source: PLoS Med. 2020 May 20;17(5):e1003120. doi: 10.1371/journal.pmed.1003120 (PMC7239392; doi:10.1371/journal.pmed.1003120)
Supplement: S1 Text — (DOCX) [file pmed.1003120.s023.docx]

**S1 Text.** PROSPERO registry record.

(Also available from PROSPERO’s website, record ID 146405; <https://www.crd.york.ac.uk/prospero/display_record.php?ID=CRD42020146405>).

| **PROSPERO** |
| --- |
| **International prospective register of systematic reviews**  Impacts of sugary drink warnings: a systematic review and meta-analysis of randomized experiments *Anna Grummon, Marissa Hall*  Citation  Anna Grummon, Marissa Hall. Impacts of sugary drink warnings: a systematic review and meta- analysis of randomized experiments. PROSPERO 2020 CRD42020146405 Available from: https://www.crd.york.ac.uk/prospero/display_record.php?ID=CRD42020146405  Review question  Across the body of experimental studies, what are the effects of sugary drink warnings compared with control conditions?  Searches  The current systematic review and meta-analysis will be conducted in accordance with PRISMA guidelines. To conduct the search, we will partner with an academic research librarian to develop and implement a comprehensive search strategy. The search will involve two steps:  1) We will search PubMed, PsycINFO, Scopus, and Communication & Mass Media Complete using the following terms and their cognates and synonyms: beverage AND (warning OR label OR message OR claim). The search will include articles published at any time and in any language (i.e., the study does not have any exclusions based on publication date or language).  2) When full-text screening is complete, we will examine the reference sections of the final set of articles included in the review for additional relevant titles not captured by the initial search. We implemented an initial database search on June 21, 2019. We then updated/repeated the search on October 25, 2019. We completed screening of records identified through database searches on November 14, 2019 and completed screening of records identified from reference lists on December 13, 2019.  Types of study to be included  Studies must use a randomized design. Both within-subjects and between-subjects randomized studies will be eligible. All other designs (e.g., quasi-experiments, case studies, trend studies, reviews and meta- analyses, commentaries, briefs, observational studies) will be excluded. Studies must be peer-reviewed and be available in (or translatable to) English.  Condition or domain being studied  This review assesses the impact of warnings on sugary drinks. We provide definitions of "warnings" and "sugary drinks" below.  Participants/population  Eligible populations are participants of any age and from any country. We will exclude studies not conducted with humans.  Intervention(s), exposure(s)  Health or nutrient warnings on sugary drinks. Health warnings are defined as direct statements about health harms of consuming a nutrient or product. Nutrient warnings are defined as interpretative messages the alert consumers that a product has a high amount of a harmful nutrient (e.g., sugar, salt, fat, saturated fat, trans fat, calories) using a "High in," "Excess of" or "WARNING" statement or the equivalent in another language. Warnings may be text-only or include an icon or photograph. Eligible warnings are shown on the front-of- package, at the point of sale, or by themselves but intended for display on a product package or at the point of sale. We will exclude warnings shown on advertisements.  Sugary drinks are defined as sodas, sports drinks, fruit drinks, sweetened coffees or teas, energy drinks, other beverages with added caloric sweetener, juice, and sweetened milk. Warnings on other products (food, alcohol, tobacco) will be excluded. Other label types (traffic light labels, health claims, star ratings, etc.) will be excluded.  Comparator(s)/control  The following comparators will be considered relevant: no label control; neutral image or message (e.g., a barcode image, a message about littering); calorie or nutrient content labels without any interpretative messaging (i.e., labels that display calorie or nutrient content [e.g., XXX calories] but do not include any additional interpretative information). Excluded comparators are traffic light labels, star ratings labels (e.g., Guiding Stars, Health Star Rating), any type of warning, and other interpretative health or nutrition labels.  Context  Main outcome(s)  In an attempt to be comprehensive in our discussion of this literature, we will quantitatively synthesize any outcome we can meta-analyze (i.e., any outcome that is measured in at least two studies), similar to Noar et al. (2015), doi:10.1136/tobaccocontrol-2014-051978. We anticipate these outcomes may include behaviors, intentions, hypothetical product selections, attitudes/beliefs, and warning reactions (e.g., attention/noticing, elaboration, emotions).  Our pre-specified primary outcome (assuming we have sufficient studies to meta-analyze this outcome) is real-stakes sugary drink purchase or consumption behavior. Real-stakes behavior will be defined as participants' non-hypothetical purchase or consumption behaviors (e.g., % buying an SSB in a shopping task, volume of SSBs consumed in the past day). To be eligible for inclusion, real-stakes behaviors can be objectively measured (e.g., purchases as recorded by the researcher) or self-reported (e.g., self-reported beverage consumption).  Relevance of this outcome to health: Sugary drink consumption has been associated with several health outcomes including weight gain, obesity, type 2 diabetes, and heart-disease. If warnings reduce sugary drink purchase or consumption behavior, this would suggest warning policies could reduce sugary drink consumption and the associated health harms, and would indicate policymakers should consider implementing policies requiring sugary drink warnings as a strategy for addressing diet-related disease.  * Measures of effect  Outcomes can be measured at any time point after exposure to intervention or comparator conditions. We will quantitatively synthesize outcomes measured on different scales by converting warnings' impacts into standardized effect sizes (Cohen's d with Hedge's correction, see details below).  Additional outcome(s)  Not applicable. As noted above, we will quantitatively synthesize any outcome we can meta-analyze (i.e., any outcome that is measured in at least two studies). In addition to real-stakes behaviors (see above), we anticipate these outcomes may include intentions, hypothetical product selections, attitudes/beliefs, and warning reactions such as attention/noticing, emotional responses and elaboration.  * Measures of effect  See above.  Data extraction (selection and coding)  Article selection will involve four steps:  1) Two independent coders will double-screen titles using the reference software Zotero. Any record included by at least one coder will be retained for abstract review.  2) Two independent coders will then double-screen all abstracts using the online software Covidence. Discrepancies in abstract screening will be resolved by a third independent reviewer.  3) Two independent coders will double-screen all full-text articles. Discrepancies in full-text screening will be resolved by a third independent reviewer.  4) One coder will review the reference lists of the included full-text articles identified in step 3 (see also "Searches" section above) to identify potentially relevant titles not screened previously. Two independent coders will screen these titles; any title included by at least one coder will be retained for further review following steps 2-3 above.  Article coding will be completed by two independent coders double-extracting all fields. The study principal investigators will develop an extraction tool in Excel. Data to extract will include information on study's: author, year, country, sample characteristics (e.g., mean age), type of randomization, number of exposure sessions, exposure setting for warnings/products (e.g., online survey), exposure medium for warnings (e.g., front-of-package), characteristics of each warning condition (e.g., warning topic) and characteristics of each control condition (e.g., type of control). The coders will pilot test the tool with a small sample of included full text articles (to comprise 10-20% of the final set of included full text articles). The coders and principal investigators will then review the pilot extraction for discrepancies among coders. We will resolve discrepancies by discussion and will refine the extraction tool to clarify definitions as needed. Then, two independent coders will double-extract the remaining full texts with the finalized extraction tool. Discrepancies in extraction will then be resolved by a third independent reviewer.  Risk of bias (quality) assessment  This review and meta-analysis focuses on experimental studies. We are therefore excluding studies that have high risk of bias to internal validity due to non-random assignment to study conditions. We will assess the following characteristics relevant to study quality: whether studies are between- vs. within-subjects experiments; sample size; whether studies are longitudinal; and whether studies assess a non-hypothetical (real stakes) behavioral outcome.  Strategy for data synthesis  We will describe and summarize findings of included studies to address the study objective. We will provide a brief narrative synthesis, describing interventions (warnings), participants, study characteristics, and effects of eligible interventions on outcomes specified above. Then, we will meta-analyze (i.e., quantitatively synthesize) any outcome measured in at least two studies, provided there is sufficient homogeneity in exposure/outcome measures among studies and sufficient statistical information is available to calculate standardized effect sizes.  Effect estimates will be independently double-extracted from included studies by the two PIs, with discrepancies resolved by consensus. Before conducting meta-analyses, we will convert all effect estimates into standardized effect sizes (Cohen's d) using the formulas provided by David B. Wilson (https://cebcp.org/practical-meta-analysis-effect-size-calculator/). When studies report multiple effect estimates for the same outcome (e.g., two measures of effects of warnings on intentions), we will combine effect sizes prior to analysis following methods described in Bornstein, Hedges, Higgins, & Rothstein (2009, ISBN: 978-0-470-05724-7). We will apply Hedges’s correction to the extracted Cohen's d's, using the formulae provided in Bornstein et al. (2009, ISBN: 978-0-470-05724-7). The Cohen's d's with Hedges's corrections for individual studies will be combined using random effects meta-analysis, implemented using Stata version 16's -meta- package. For each meta-analyzed outcome, we will report the mean weighted effect size as well as its 95% confidence interval (CI), interpreting CIs that do not overlap 0 as statistically significant effects. We will assess heterogeneity using the I2 and Q statistics.  Analysis of subgroups or subsets  We plan to conduct moderator analysis for the following outcomes provided these outcomes have both significant heterogeneity and an adequate number of studies (at least 2 studies per level of the categorical moderator): real-stakes behaviors, intentions, hypothetical selections/behavior, perceived likelihood, and healthfulness perceptions. We selected these outcomes based on their relevance to policymaking. We plan to examine the following potential categorical moderators as long as there are sufficient studies to conduct moderation/subset analyses: warning topic; type of randomization; setting in which warnings were displayed (warning displayed on actual product in person vs. some other setting); sugary drink consumer status of sample; ages included in sample. We will calculate effect sizes and 95% confidence intervals for each level of the moderating variable and will assess heterogeneity in those effect sizes using the Qb statistic.  Contact details for further information  Anna H Grummon annagrummon@gmail.com  Organisational affiliation of the review  Harvard TH Chan School of Public Health  Review team members and their organisational affiliations  Dr Anna Grummon. Harvard TH Chan School of Public Health Dr Marissa Hall. University of North Carolina, Chapel Hill  Type and method of review  Meta-analysis, Systematic review  Anticipated or actual start date  01 June 2019  Anticipated completion date  30 June 2020  Funding sources/sponsors  Robert Wood Johnson Foundation Healthy Eating Research (no number).  Conflicts of interest Language  English  Country  United States of America  Stage of review  Review Ongoing  Subject index terms status  Subject indexing assigned by CRD  Subject index terms  Beverages; Dietary Supplements; Humans  Date of registration in PROSPERO  04 February 2020  Date of publication of this version  04 February 2020  Details of any existing review of the same topic by the same authors Stage of review at time of this submission  NA  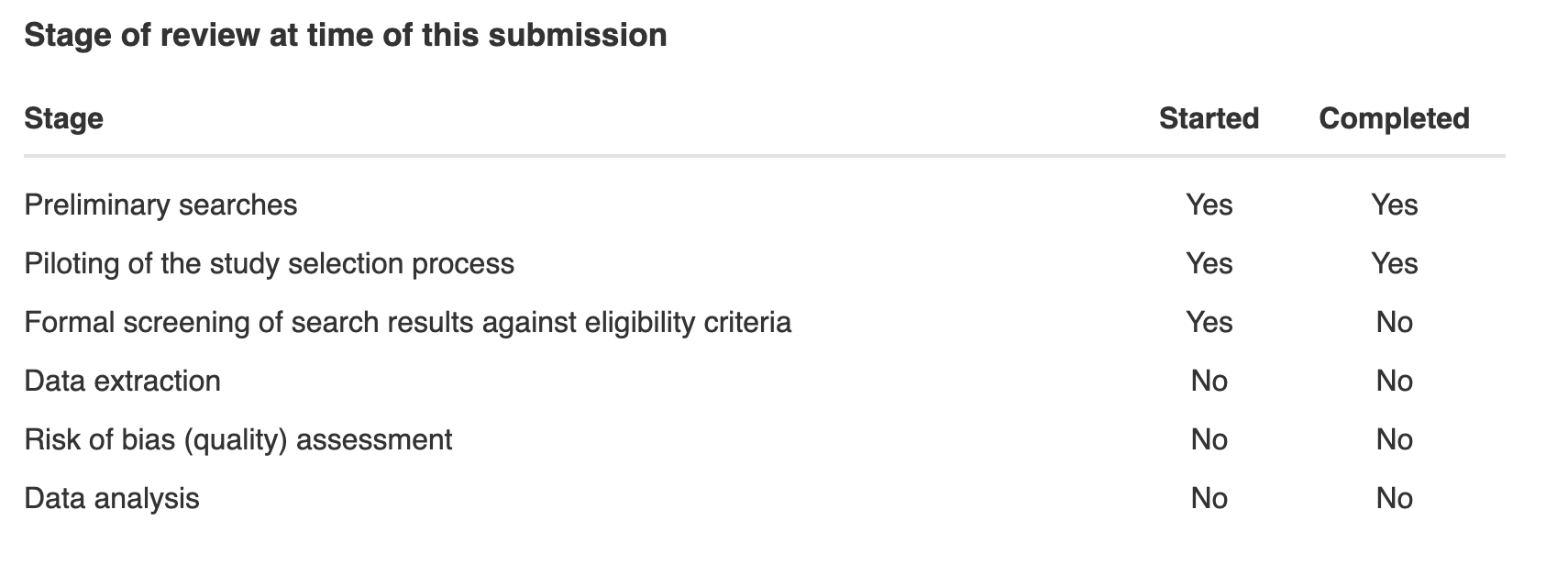 |
